# Supplementary material for: Uncovering supramolecular chirality codes for the design of tunable biomaterials
Source: Nat Commun. 2024 Jan 26;15:788. doi: 10.1038/s41467-024-45019-2 (PMC10817930; doi:10.1038/s41467-024-45019-2)
Supplement: Supplementary file 1 — Supplementary Information [file 41467_2024_45019_MOESM1_ESM.pdf]

Supplementary Information

Table of Contents:

Supplementary Note 1. Chirality Definitions and Terminology……………………………………………..……..3

Supplementary Figure 1. KLVFFAE Synthesis……………………………………………..……..…..………4

Supplementary Figure 2. KLVFFAL Synthesis………………………………………………………..…...…. 4

Supplementary Figure 3. KLVFFAV Synthesis………………………………..……...…..…………….…..5

Supplementary Figure 4. REDOR NMR of KLVFFA(E), L, and V Nanotubes…………………………..….5

Supplementary Figure 5. SAXS of KLVFFAL and KLVFFAV Nanotubes…………….…………………..…6

Supplementary Figure 6. XRD of KLVFFAE, KLVFFAL, and KLVFFAV Nanotubes……………………..…..6

Supplementary Figure 7. CD of KLVFFAL and KLVFFAV Nanotubes………………………………………7

Supplementary Figure 8. TEM of Co-assembled KLVFFAL and KLVFFAV……………………………..….7

Supplementary Figure 9. CD of Co-assembled KLVFFAL and KLVFFAV…………………………….…....8

Supplementary Figure 10. REDOR NMR of Co-assembled KLVFFAL and KLVFFAV……………………..…..8

Supplementary Figure 11. KLVFFA-PEG_2 Synthesis………………………..……………………………......9

Supplementary Figure 12. CD Spectra of KLVFFA-(R) Peptides in HFIP……………………..………..….9

Supplementary Figure 13. Slow Cooling CD of KLVFFAV in HFIP………………………………..……….10

Supplementary Figure 14. Fast Cooling KLVFFAV in HFIP…………………………………………….…..10

Supplementary Figure 15. TEM of KLVFFAE and KLVFFAV Heated in ACN…………….……………..….11

Supplementary Figure 16. CD of KLVFFAE and KLVFFAV Heated in ACN……………………….……..11

Supplementary Figure 17. KFFFF-PEG_2 Synthesis …………………………..…………………………...…..12

Supplementary Figure 18. H_2N-FFFF-PEG_2 Synthesis……………………..…...………………………….12

Supplementary Figure 19. Fmoc-FFFF-PEG_2 Synthesis…………………………..……..……………...13

Supplementary Figure 20. Synthesis of Fmoc-FFFF-PEG_2 (D amino acids)………………… ……...…..13

Supplementary Figure 21. Assembly of Fmoc-FFFF-PEG_2 (D amino acids)……….. …………….……....14

Supplementary Figure 22. Fmoc-FFF-PEG_2 Synthesis….......……………………………………….….....14

Supplementary Figure 23. Fmoc-FFF-PEG_2 CD and TEM....……………………………… ……..….......15

Supplementary Figure 24. Fmoc-FF-PEG_2 Synthesis…………………………………………………...….15

Supplementary Figure 25. Fmoc-FF-PEG_2 CD and TEM…………………………..………….…..….16

Supplementary Figure 26. Fmoc-FFFF-NH_2 Synthesis…………………………………….……........16

Supplementary Figure 27. Fmoc-FFFF-NH_2 CD and TEM………………………………..…...............17

Supplementary Figure 28. Fmoc-FFFF-PEG_2-Kaz Synthesis…………………………………..…...........17

Supplementary Figure 29. Fmoc-FFFF-PEG$_2$-Cys Synthesis……………………..…………………..……….18

Supplementary Figure 30. SEM of Chiral Supramolecular Ribbons……………………………………….18

Supplementary Figure 31. Melting CD Spectra………..…………………......……………………….…...19

Supplementary Figure 32. Fmoc-4F-PEG$_2$-Cys Melting With TCEP……………………………...…..19

Supplementary Figure 33. Temperature Dependent Pitch of Fmoc-4F-PEG$_2$……....…….…………….………19

Supplementary Figure 34. Fmoc-4F-PEG$_2$ Ribbon Thickness……………………….….………………...20

Supplementary Figure 35. Bilayer Assembly of Fmoc-4F-PEG$_2$ …………………….….….……….……....20

Supplementary Figure 36. FTIR of Aged and Annealed Fmoc-4F-PEG$_2$……...….………………...…....21

Supplementary Figure 37. Length of Nucleated Fmoc-4F-PEG$_2$……………......…..…..………….……….21

Supplementary Figure 38. Fmoc-4F-PEG$_2$ Structure Transition CLSM..……..... ……….…………….....22

Supplementary Figure 39. Representative FRAP Images of Fmoc-4F-PEG$_2$ ....….….………….. ……….....23

Supplementary Figure 40. FRAP Quantification of Fmoc-4F-PEG$_2$ …...……....……………….………......24

Supplementary Figure 41. Fluorescamine Standard Curve…………..…………..………..……..……25

Supplementary Figure 42. Degradation MS 27 min……………..……..……………...………..………...25

Supplementary Figure 43. Degradation MS 28 min……………………………………………….………..…. 26

Supplementary Figure 44. Degradation MS 35 min…………………………..………………...……...26

Supplementary Figure 45. Degradation MS 46 min………………………………………….………….…...27

Supplementary Figure 46. Chymotrypsin Activity Assay………………………………………………….. 27

Supplementary Figure 47. DOX Loading for FFFF-PEG$_2$……………………………………………...……..28

Supplementary Figure 48. CLSM of DOX Release……………………………………………...…………..28

Supplementary Figure 49. DOX Binding to Left- and Right-Handed Structures……………………………29

Supplementary Figure 50. DOX Calibration Curve for HeLa Cells……………………………..…………....29

Supplementary Figure 51. DOX Calibration Curve for MB231 Cells…………………………………...…..30

Supplementary Figure 52. Cell Viability at 24h……………………………………….……………………..30

Supplementary Figure 53. Peptide Only Cell Viability Controls…………………………………………..….31

Supplementary Figure 54. Effective Dose 24h and 48h Comparison…………………………..……….…..31

Supplementary Notes

Supplementary Note 1: Chirality Definitions and Terminology

Chirality is defined in various ways across several length scales in biomolecules. On the molecular level, amino acids are defined as (D) or (L) depending on the way in which they rotate planar light. D amino acids will rotate light to the right while L amino acids which make up the proteins in our body will rotate light to the left. As chirality extends beyond the single molecule level into secondary protein structure, it is often described as left- or right-handed for each structure such as helices or b-sheets. However, b-sheet the chirality can be defined laterally with respect to a single peptide strand or axially with respect to the entire b-sheet entity and these handedness assignments are not necessarily the same. For the context of this paper, we will use the axial definition to describe the handedness of b-sheets; left-handed sheets will have a negative cotton effect in the circular dichroism peak near 220 nm and right-handed sheets will have a positive cotton effect in that region. As these structures assemble further into supramolecular materials, chiral twists are defined as either M-type (left) or P-type (right) helices with the handedness determined by SEM and TEM images. We correlate negative-to-positive cotton effect on CD with supramolecular chirality inversion allowing us to spectroscopically track supramolecular twist.

Supplementary Figures

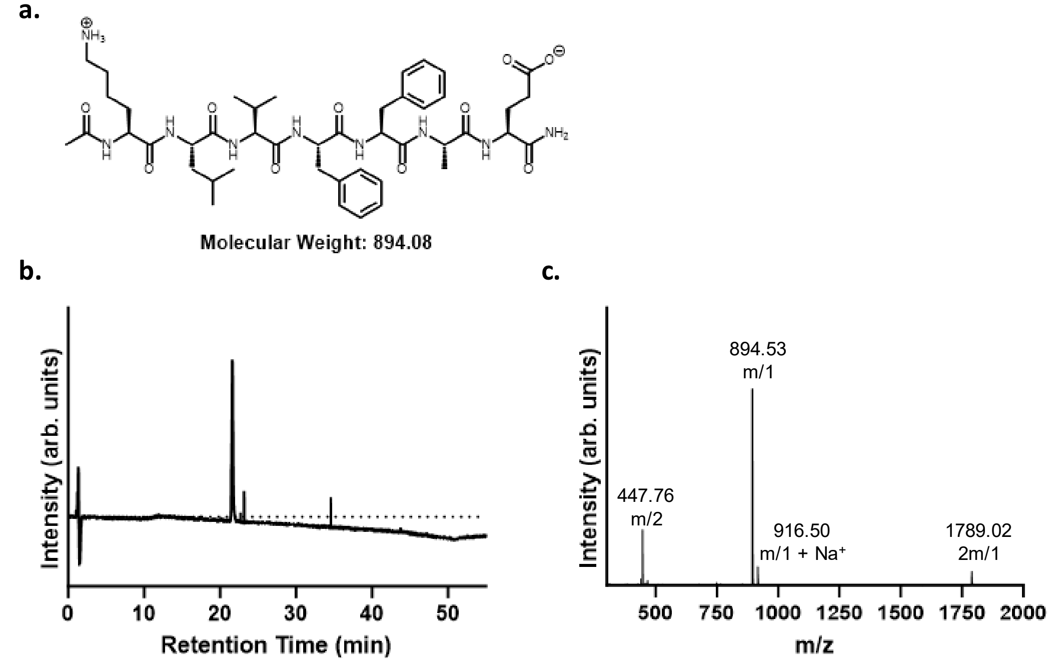

Supplementary Figure 1. KLVFFAE Synthesis. Chemical structure (a) of the KLVFFAE peptide. Analytical HPLC trace monitoring peptide absorbance at 214 nm (b) to confirm peptide purity and ESI mass spectrometry (c) confirming peptide identity.

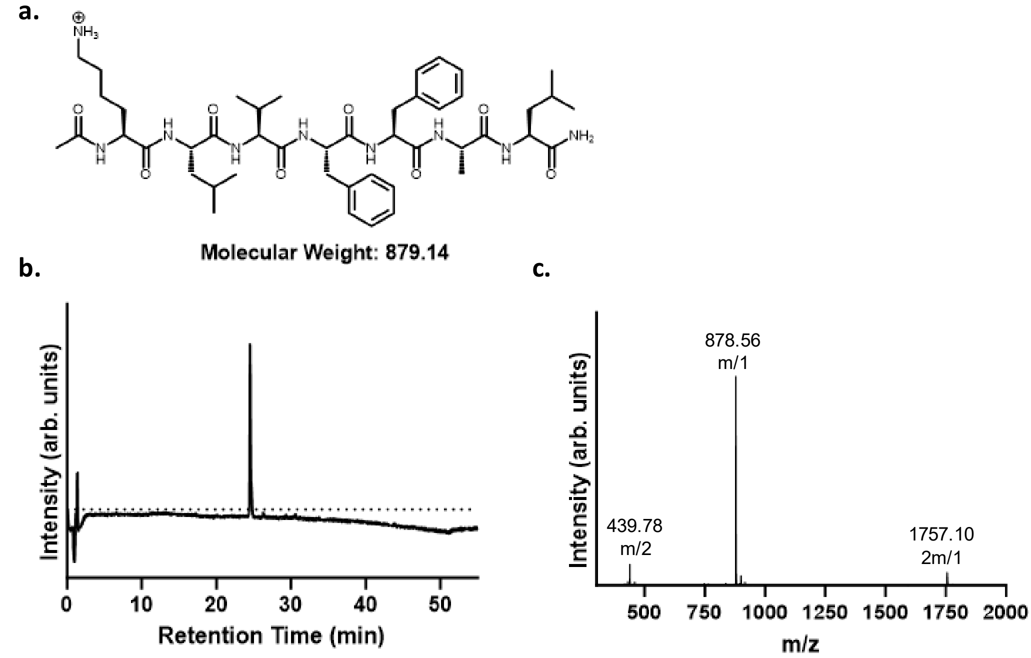

Supplementary Figure 2. KLVFFAL Synthesis. Chemical structure (a) of the KLVFFAL peptide. Analytical HPLC trace monitoring peptide absorbance at 214 nm (b) to confirm peptide purity and ESI mass spectrometry (c) confirming peptide identity.

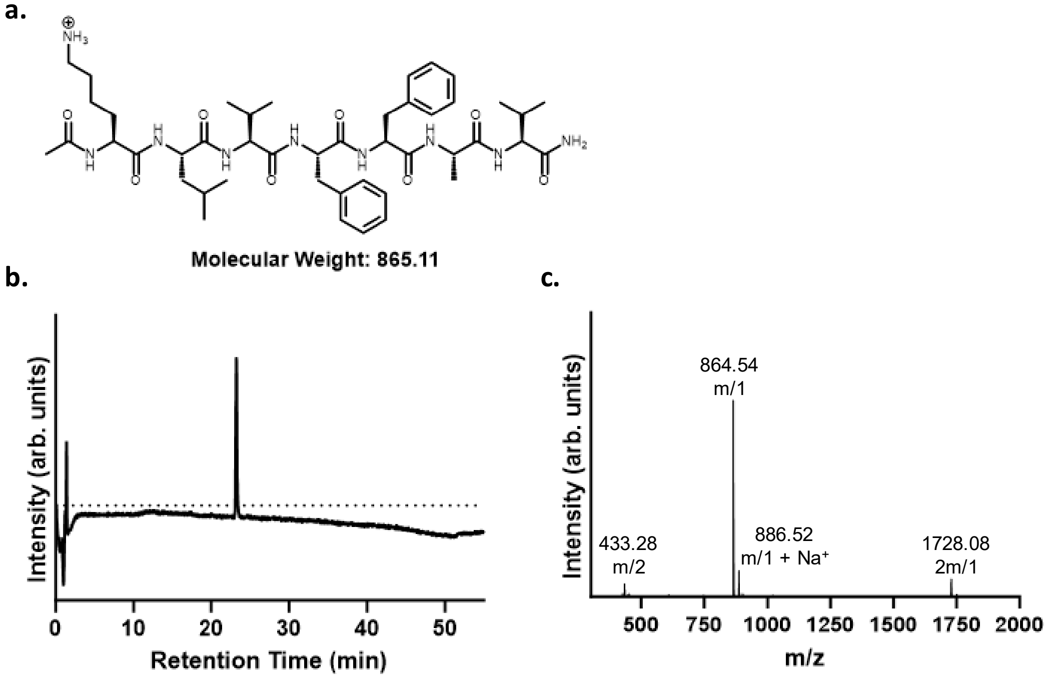

Supplementary Figure 3. KLVFFAV Synthesis. Chemical structure (a) of the E22V peptide. Analytical HPLC trace monitoring peptide absorbance at 214 nm (b) to confirm peptide purity and ESI mass spectrometry (c) confirming peptide identity.

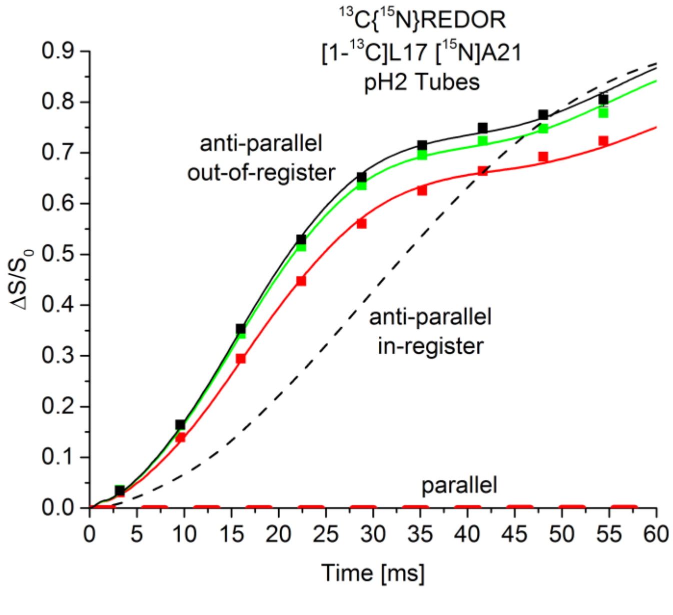

Supplementary Figure 4. REDOR NMR of KLVFFA(E), L, and V Nanotubes. $^{13}C\{^{15}N\}$REDOR dephasing of [1-^{13}C]V18 [^{15}N]A21 Ac-KLVFFAE-NH_2 (black), Ac-KLVFFAL-NH_2 (red) and Ac-KLVFFAV-NH_2 (green) peptides assembled as nanotubes. Experimental data points fit to an anti-parallel out-of-register b-sheet (solid-lines). Dashed lines are calculated REDOR dephasing for in-register anti-parallel (black) and parallel (red) b-sheets. E22L bundled tubes were not spun down, hence the lower plateau.

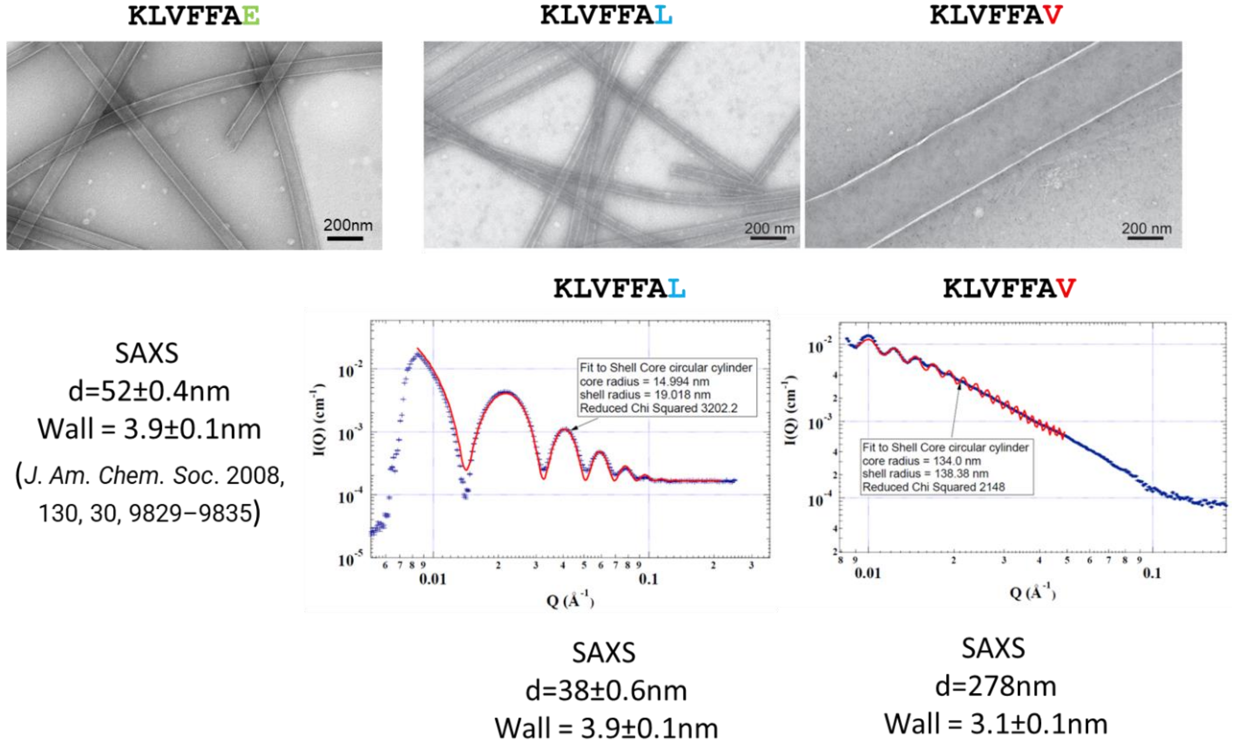

Supplementary Figure 5. SAXS of KLVFFAL and KLVFFAV Nanotubes. TEM images (top) of nanotubes assembled in 40% acetonitrile with 0.1% TFA (representative of at least three independent assemblies). SAXS (bottom) of 1.3 mM KLVFFAL and 1.3 mM KLVFFAV assembled in 40% acetonitrile with 0.1% TFA at room temperature for two weeks (blue) with best fit shown in red.

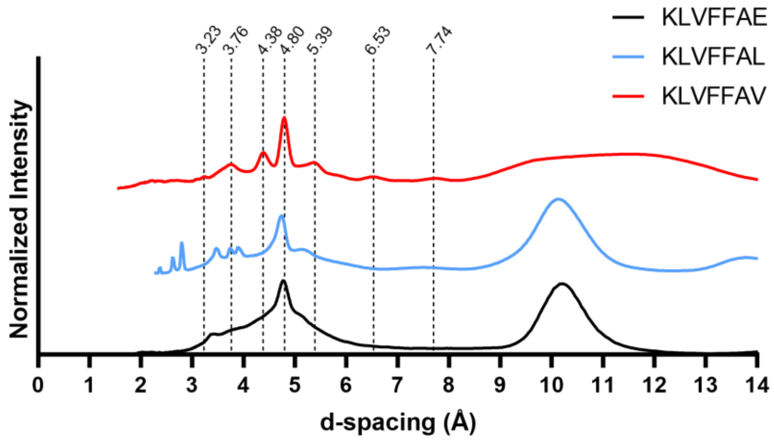

Supplementary Figure 6. XRD of KLVFFAE (black, KLVFFAL (blue) and KLVFFAV (Red) peptides assembled as nanotubes. 2nd derivative analysis of the reflections of the KLVFFAV nanotubes are indicated with dashed lines. Spectra were normalized and offset for clarity.

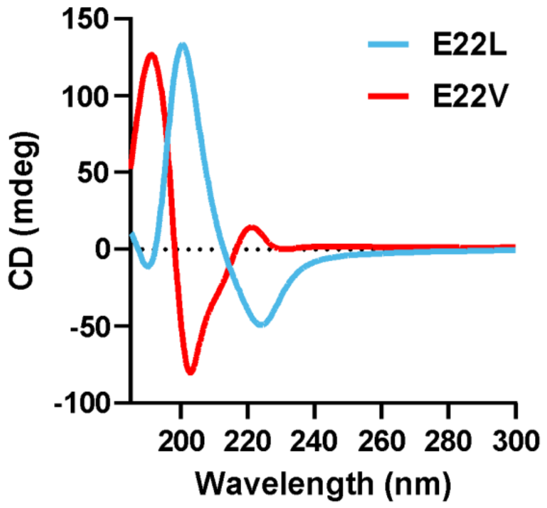

Supplementary Figure 7. CD of KLVFFAL (E22L) and KLVFFAV (E22V) Nanotubes. CD spectra of the two pure peptide assemblies are almost mirror images of each other. KLVFFAL assemblies display a characteristic β-sheet signature with a minimum at 225 nm and a maximum at 205 nm, while KLVFFAV assemblies contain a minimum at 203 nm and a positive Cotton effect at 221 nm.

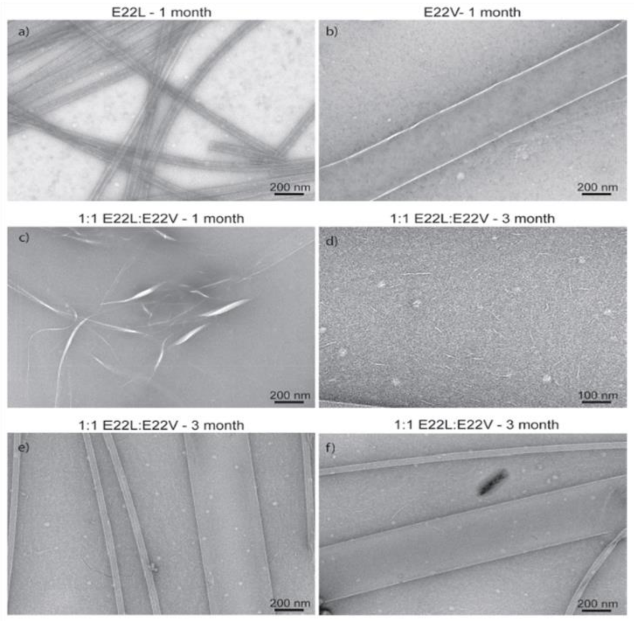

Supplementary Figure 8. TEM of Co-assembled KLVFFAL and KLVFFAV. KLVFFAL (E22L) and KLVFFAV (E22V) at 0.65 mM each in 40% acetonitrile + 0.1% TFA at 4°C and allowed to assemble for a total of 3 months. KLVFFAL (a) assembles into 40 nm diameter tubes, while KLVFFAV (b) assembled into 280 nm diameter tubes. For the 1:1 mixture, after 1 month (c) incubation only a heterogenous mixture of filaments and sheets were observed. After longer incubation (3 months), small and large tubes as well as a population of short filaments had emerged (d-f). Pure samples represent at least three individual assemblies, co-assembled samples are representative of a single sample.

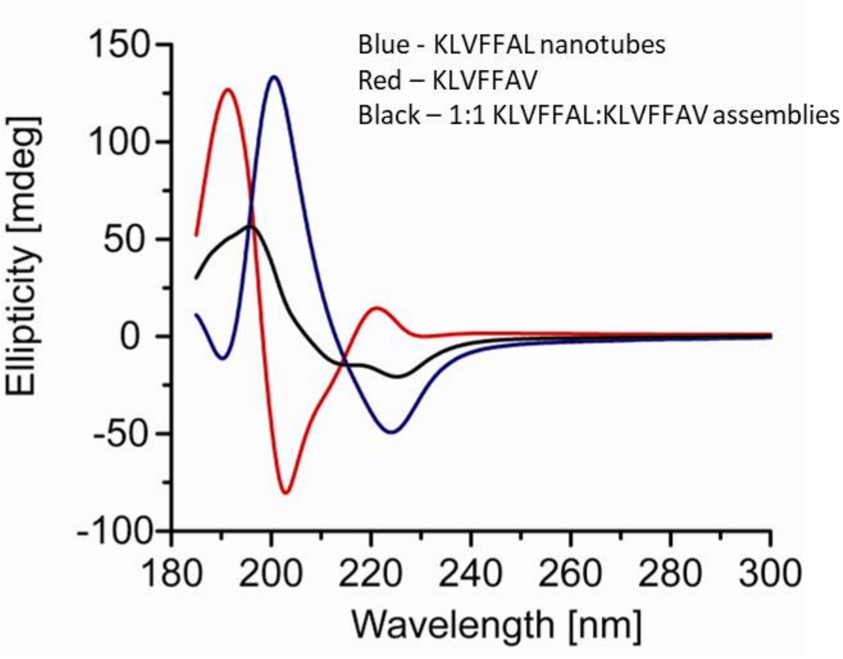

Supplementary Figure 9. CD of Co-assembled KLVFFAL and KLVFFAV. CD spectra of the two pure peptide assemblies compared to CD measurements of the 1:1 mixture which qualitatively appears to be a simple 1:1 summation of KLVFFAL and KLVFFAV CD curves (from Supplementary Figure 7).

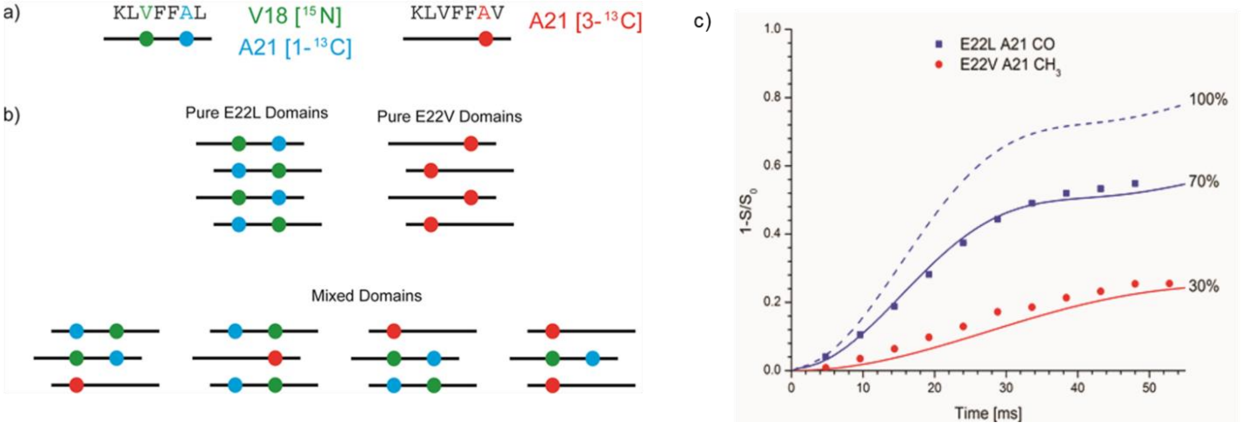

Supplementary Figure 10. $^{13}C\{^{15}N\}$REDOR NMR of Co-assembled KLVFFAL and KLVFFAV. $^{13}C\{^{15}N\}$REDOR experiment to detect the degree of mixing in the propagated assemblies from 1:1 mixtures of KLVFFAL and KLVFFAV. (a) Ac-KL[^{15}N]VFF[1-^{13}C]AL-NH_2 peptides were ^{13}C enriched at Ala 21 and ^{15}N enriched at Val 18; Ac-KLVFF[3-^{13}C]AV-NH_2 enriched in ^{13}C at Ala 21 sidechain (b) In this labeling scheme pure KLVFFAL domains can be detected as well as mixed peptide domains. Since the KLVFFAV peptide does not contain an ^{15}N, pure domains are not detected. (c) $^{13}C\{^{15}N\}$REDOR dephasing of a 1:1 mixture of KL[^{15}N]VFF[1-^{13}C]AL and KLVFF[3-^{13}C]AV assemblies. The complete 100% dephasing curve (dotted blue line) would be expected if peptides only assembled into pure E22L domains; 70% dephasing curve (solid blue line) and 30% dephasing curve (solid red line) is compared with experimental observed data for KLVFFAL's A21 CO (blue squares) and KLVFFAV's A21 CH_3 (red circles).

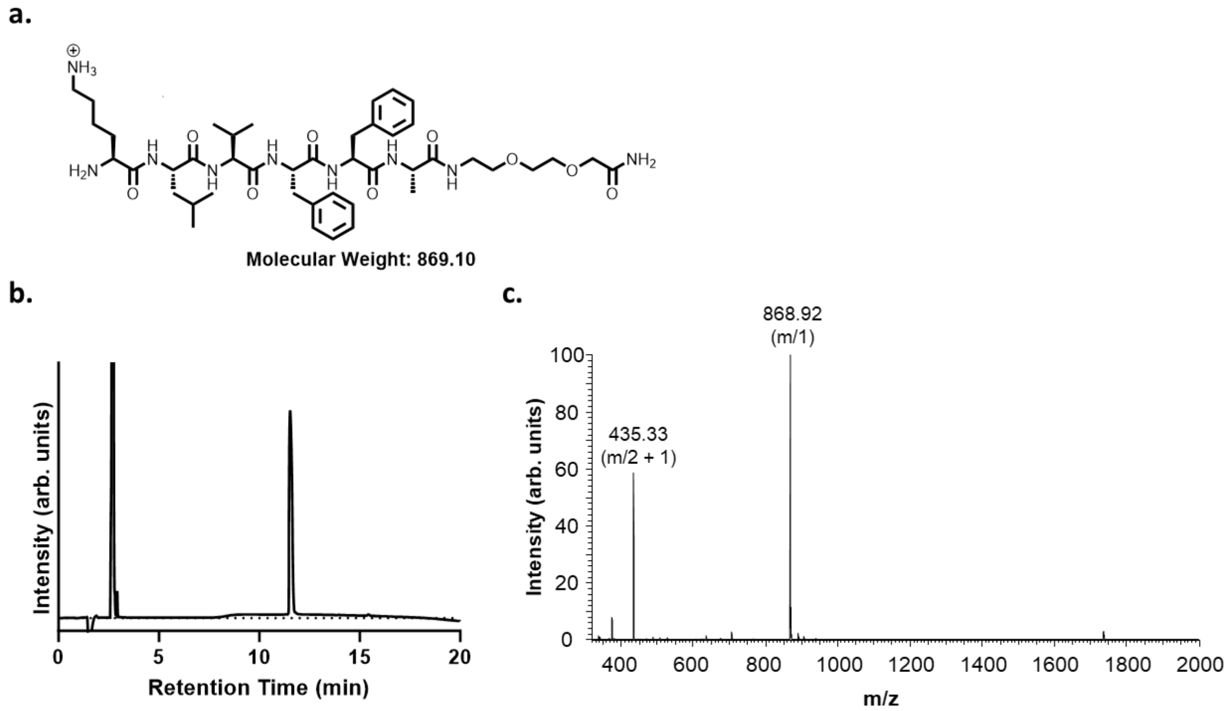

Supplementary Figure 11. KLVFFA-PEG$_2$ Synthesis. Chemical structure (a) of the KLVFFA-PEG$_2$ peptide. Analytical HPLC trace monitoring peptide absorbance at 214 nm (b) to confirm peptide purity and ESI mass spectrometry (c) confirming peptide identity.

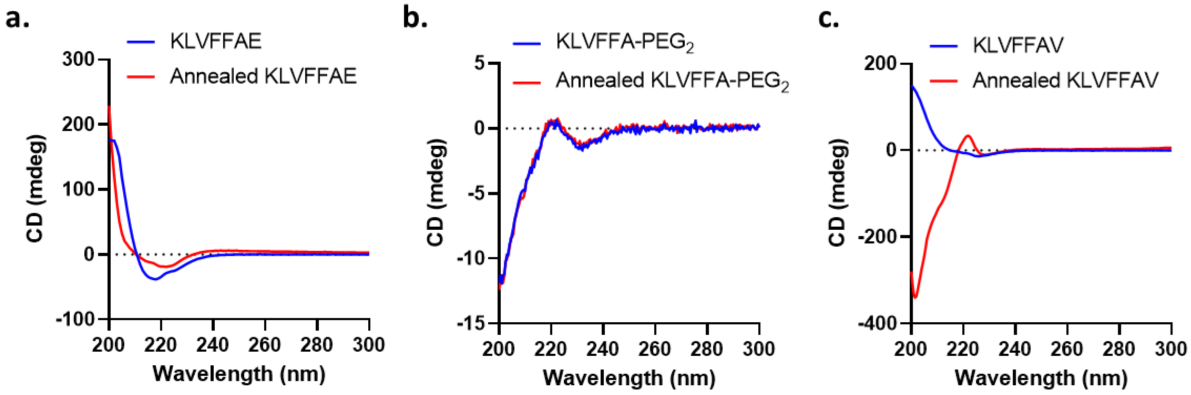

Supplementary Figure 12. CD spectra of KLVFFA-(R) peptides in HFIP. CD spectra of KLVFFAE (a), KLVFFA-PEG$_2$ (b), and KLVFFAV (c) after either aging 2h or annealing in 10% HFIP.

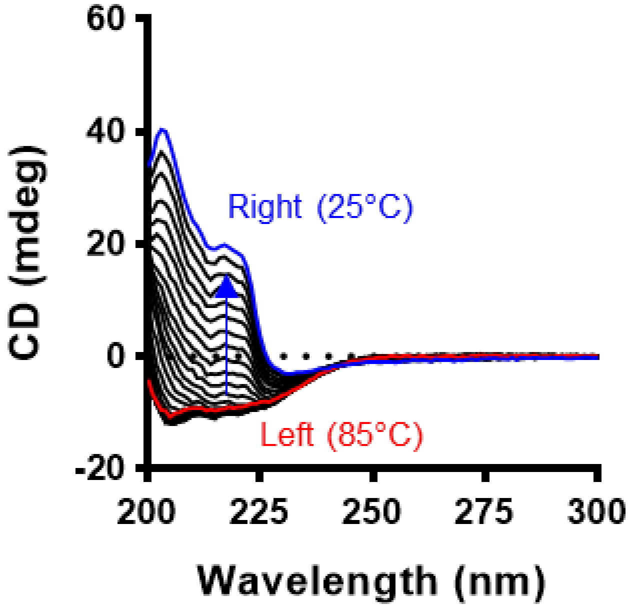

Supplementary Figure 13. Slow Cooling CD of KLVFFAV in HFIP. CD spectra of KLVFFAV during the slow cooling process (1°C/min). At 85°C, left-handed signal is present which converts to right-handed during the cooling process.

a.

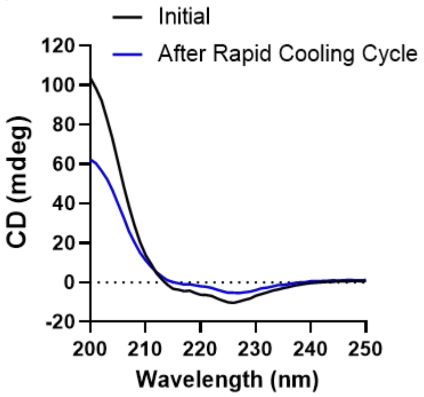

b.

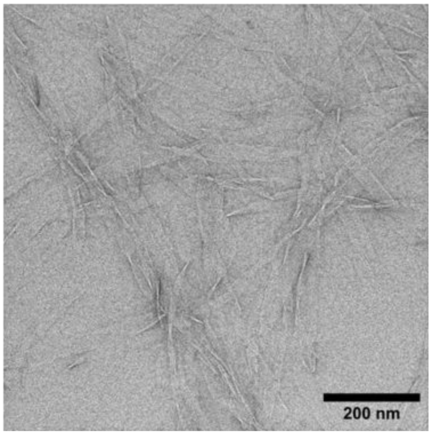

Supplementary Figure 14. Fast Cooling of KLVFFAV in HFIP. CD spectra (a) of KLVFFAV after aging 2h or after heating followed by a rapid cooling cycle. TEM (b) after the rapid cooling showing that tubes were not successfully formed (representative of two independent assemblies).

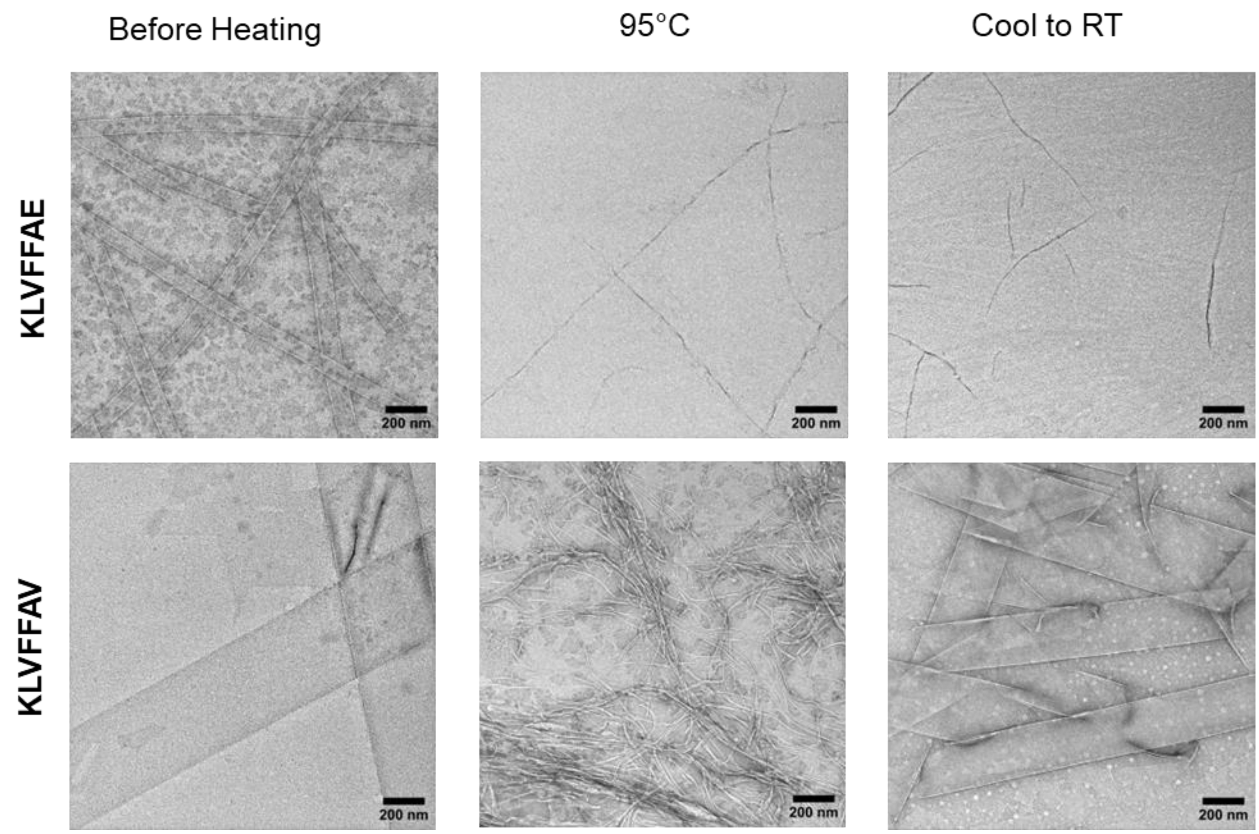

Supplementary Figure 15. TEM of Nanotubes Heated in ACN. Peptides were assembled at least two weeks in 40% Acetonitrile, 0.1% TFA at 4°C. Peptides were diluted to 260µM before being heated to 95 °C and subsequently cooled to RT. Representative images of two independent experiments.

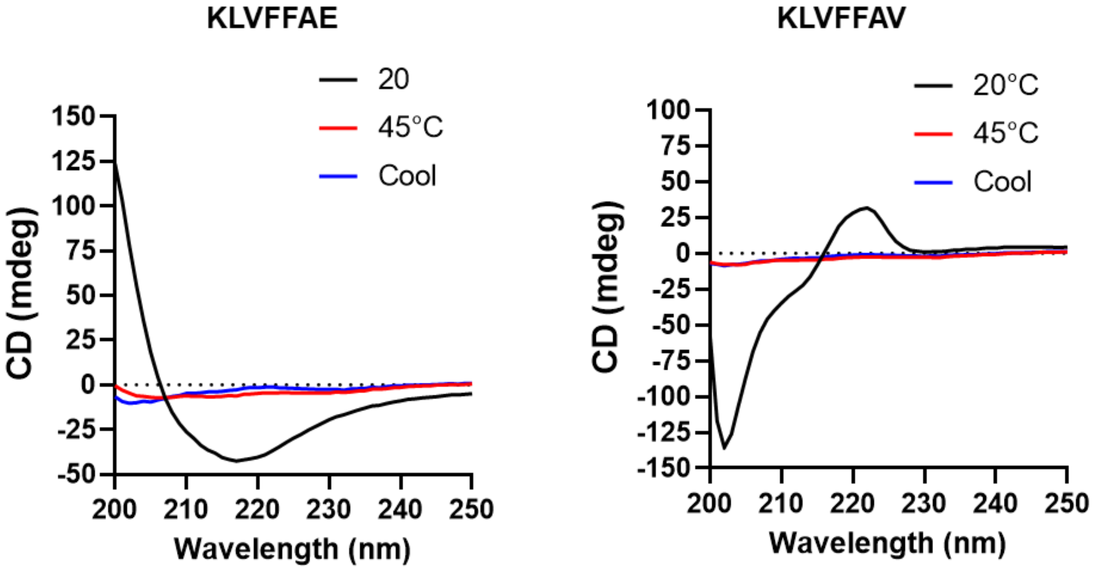

Supplementary Figure 16. CD of Nanotubes Heated in ACN. Peptides were assembled at least two weeks in 40% Acetonitrile, 0.1% TFA at 4°C. Peptides were diluted to 260µM before being heated on CD until signal stopped changing followed by subsequent cooling.

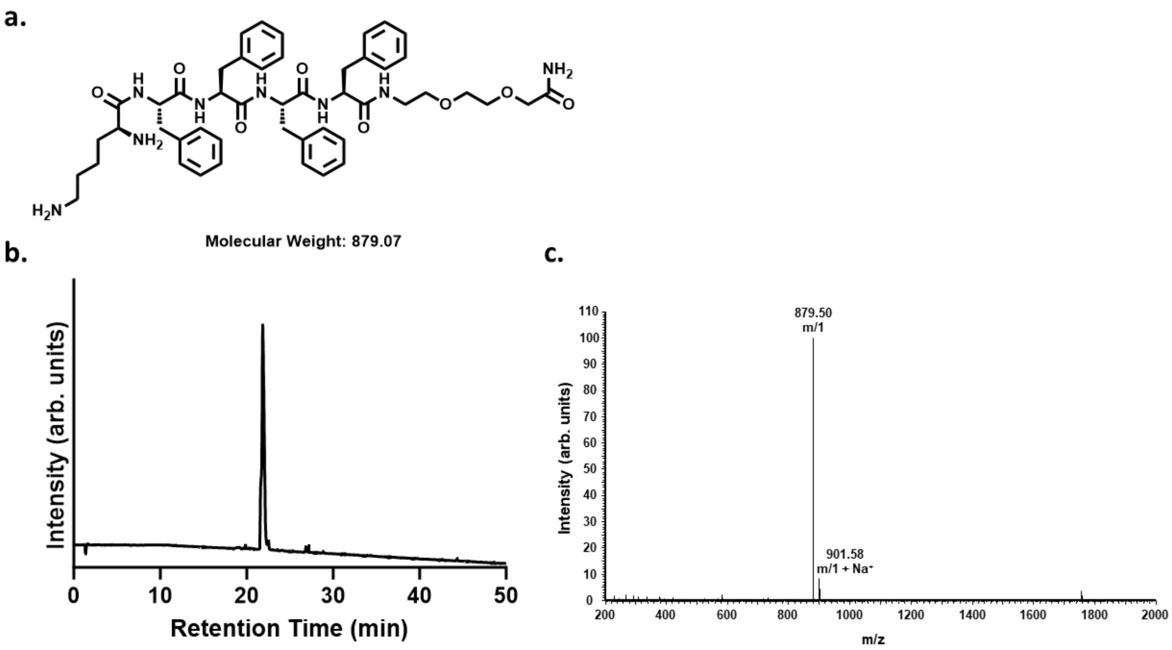

Supplementary Figure 17. KFFFF-PEG$_2$ Synthesis. Chemical structure (a) of the KFFFF-PEG$_2$ peptide. Analytical HPLC trace monitoring peptide absorbance at 214 nm (b) to confirm peptide purity and ESI mass spectrometry (c) confirming peptide identity.

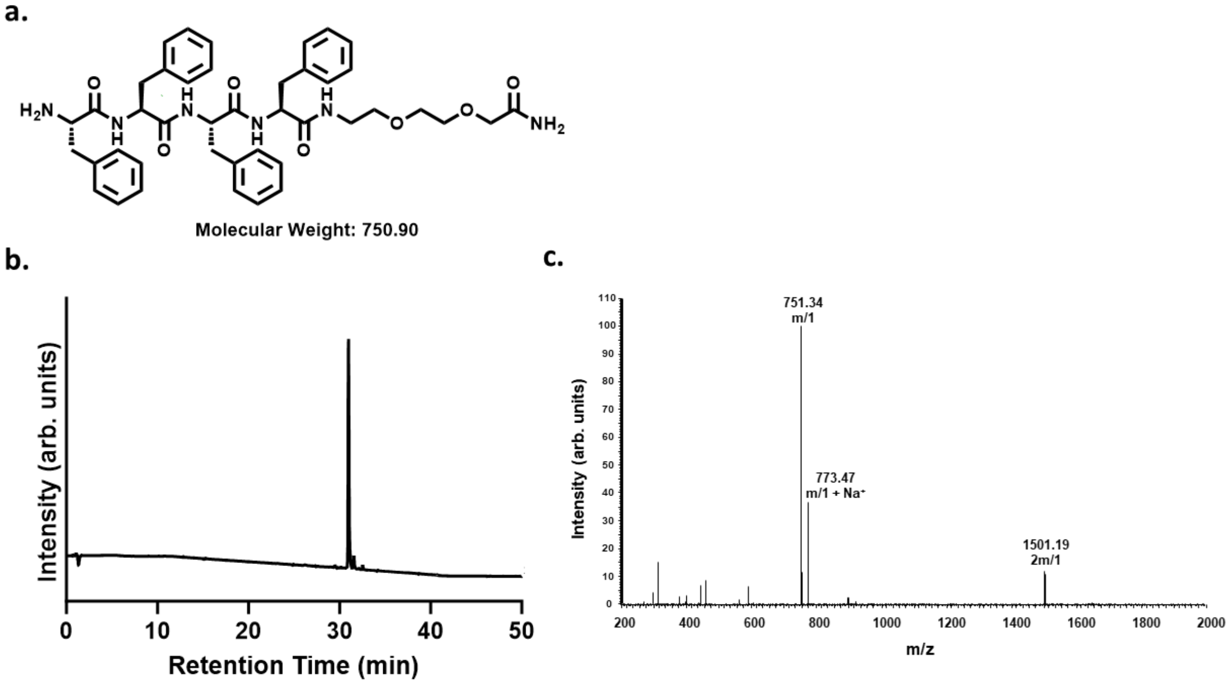

Supplementary Figure 18. H$_2$N-FFFF-PEG$_2$ Synthesis. Chemical structure (a) of the FFFF-PEG$_2$ peptide. Analytical HPLC trace monitoring peptide absorbance at 214 nm (b) to confirm peptide purity and ESI mass spectrometry (c) confirming peptide identity.

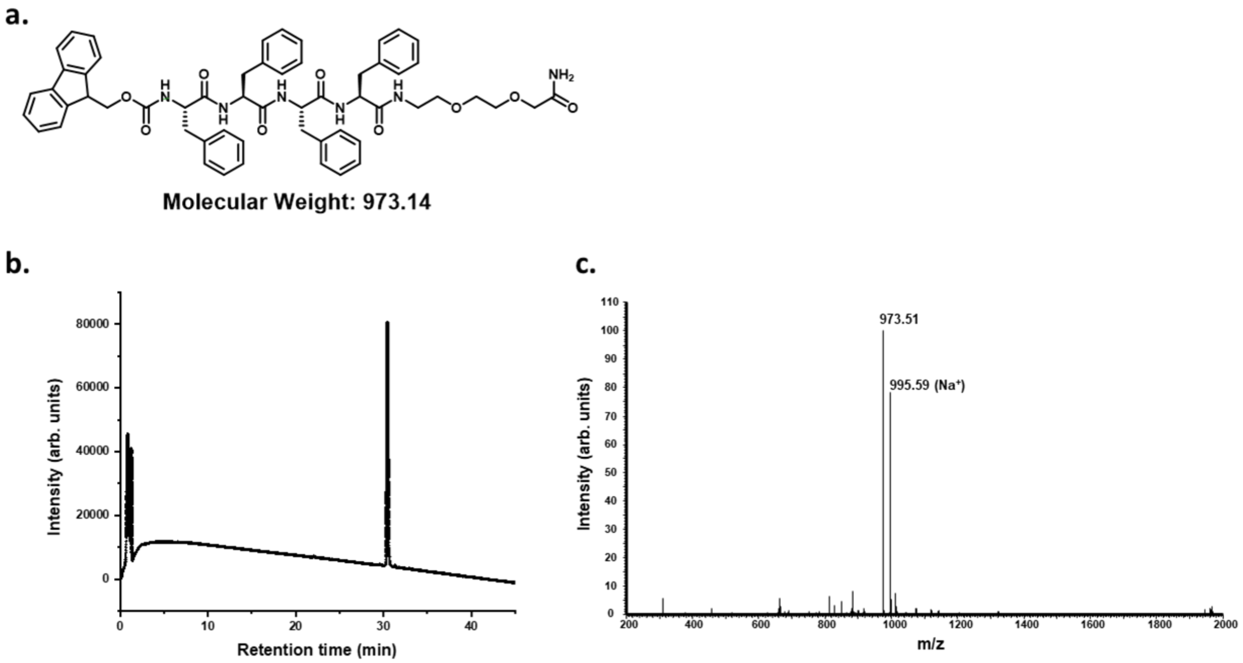

Supplementary Figure 19. Fmoc-FFFF-PEG$_2$ Synthesis. Chemical structure (a) of the Fmoc-FFFF-PEG$_2$ peptide. Analytical HPLC trace monitoring peptide absorbance at 214 nm (b) to confirm peptide purity and ESI mass spectrometry (c) confirming peptide identity.

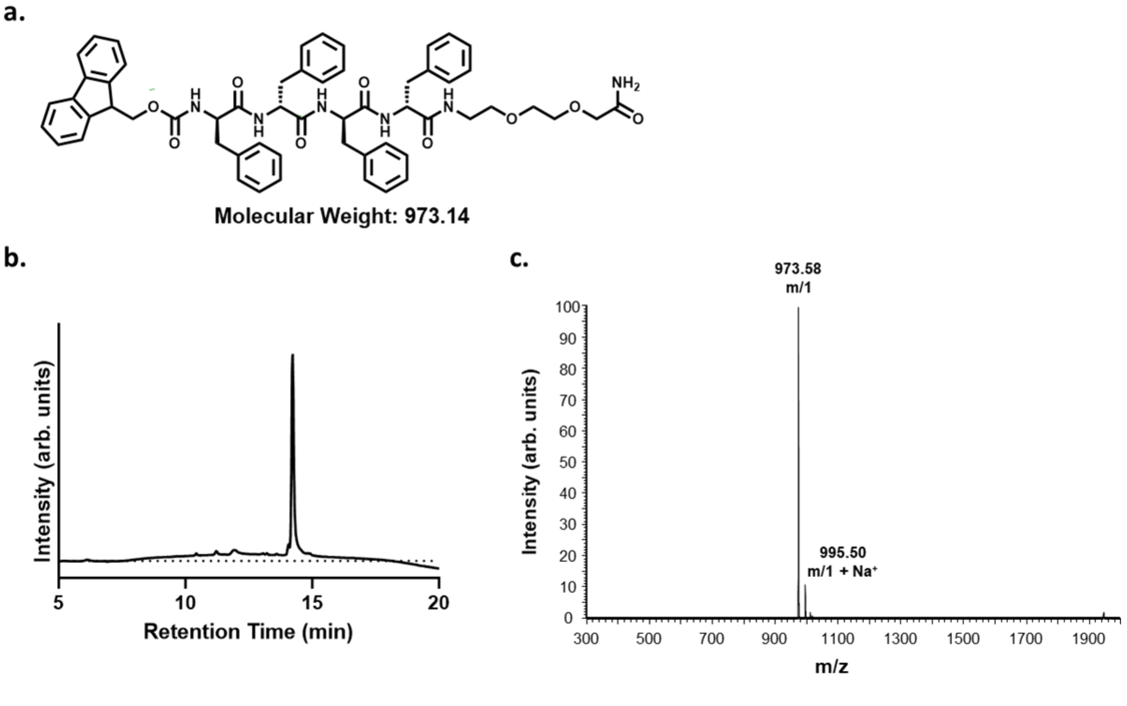

Supplementary Figure 20. Synthesis of Fmoc-FFFF-PEG$_2$ (D amino acids). Chemical structure (a) of the Fmoc-FFFF-PEG$_2$ peptide (D amino acids). Analytical HPLC trace monitoring peptide absorbance at 214 nm confirming peptide purity (b) and ESI mass spectrometry (c) confirming peptide identity.

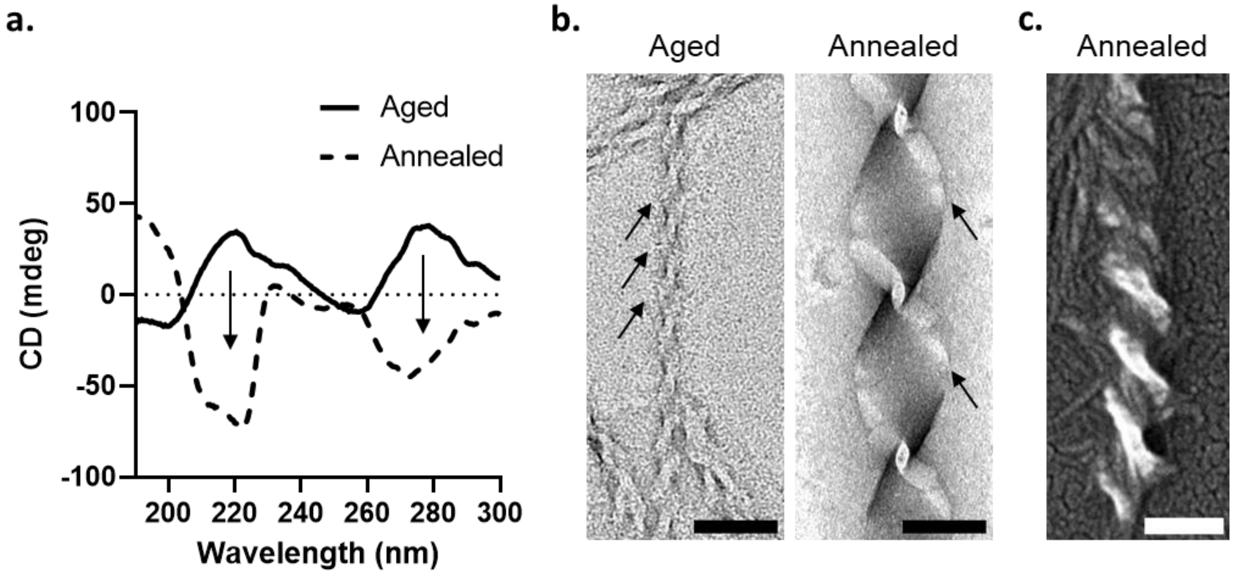

Supplementary Figure 21. Assembly of Fmoc-FFFF-PEG$_2$ (D amino acids). CD spectra (a) of the Fmoc-FFFF-PEG$_2$ peptide (D amino acids) in 10% HFIP at 250 µM. TEM images (B) showing helical twist of supramolecular ribbons (50 nm scale bars, arrows denote twist direction). SEM image (c) confirming the left-handed twist of the ribbons after annealing (100 nm scale bar). Representative images of a single experiment.

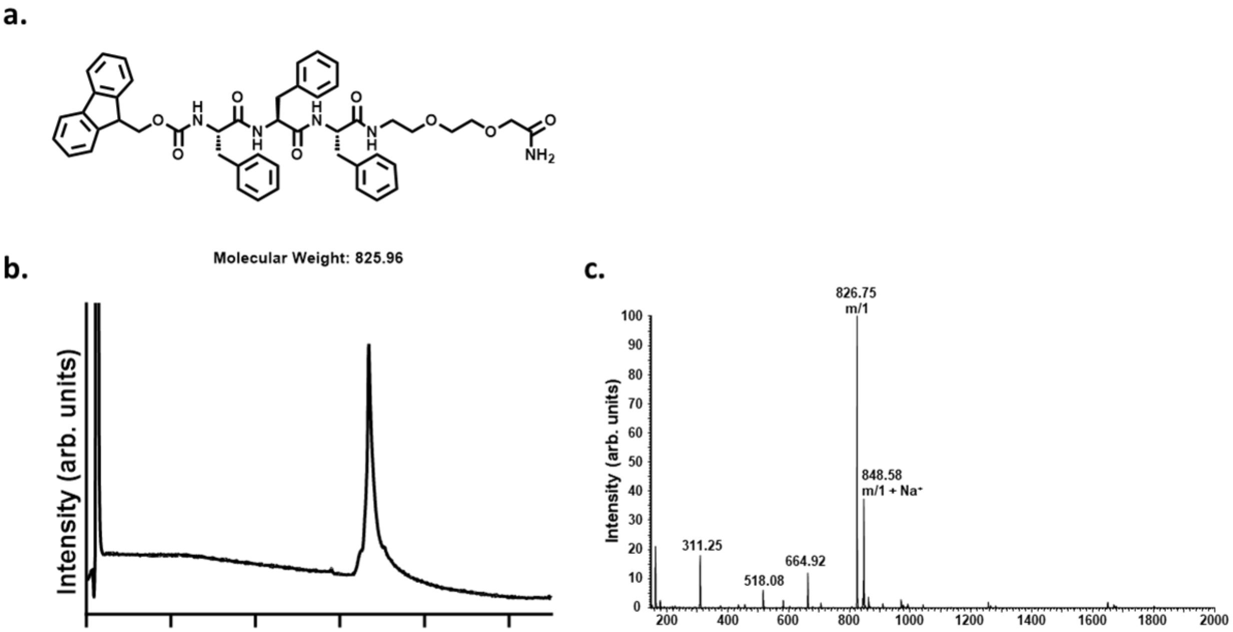

Supplementary Figure 22. Fmoc-FFF-PEG$_2$ Synthesis. Chemical structure (a) of the Fmoc-FFF-PEG$_2$ peptide. Analytical HPLC trace monitoring peptide absorbance at 214 nm (b) to confirm peptide purity and ESI mass spectrometry (c) confirming peptide identity.

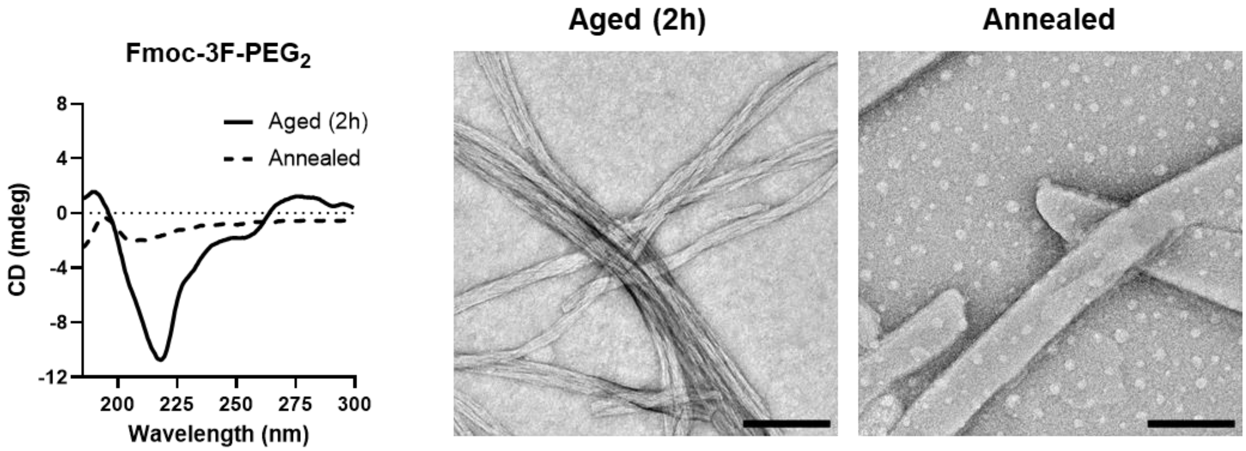

Supplementary Figure 23. Fmoc-FFF-PEG$_2$ CD and TEM. Fmoc-FFF-PEG$_2$ was assembled in 10% HFIP for either 2h at room temperature or annealed before collecting CD spectra (left) or TEM images (right), scale bars = 200 nm. Representative images of two independent assemblies.

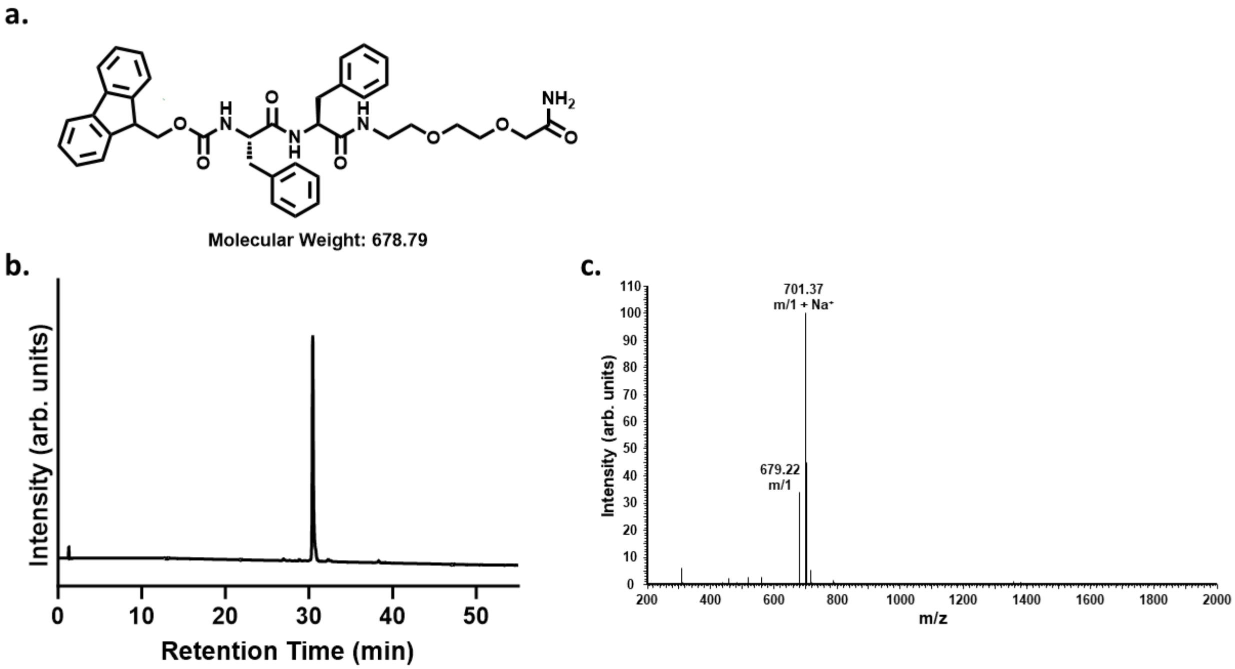

Supplementary Figure 24. Fmoc-FF-PEG$_2$ Synthesis. Chemical structure (a) of the Fmoc-FF-PEG$_2$ peptide. Analytical HPLC trace monitoring peptide absorbance at 214 nm (b) to confirm peptide purity and ESI mass spectrometry (c) confirming peptide identity.

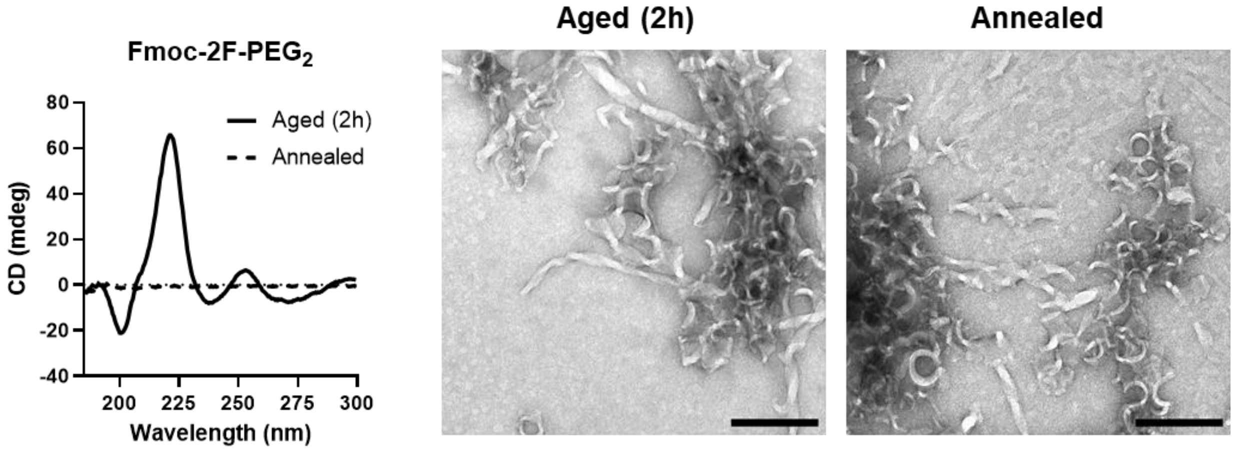

Supplementary Figure 25. Fmoc-FF-PEG$_2$ CD and TEM. Fmoc-FF-PEG$_2$ was assembled in 10% HFIP for either 2h at room temperature or annealed before collecting CD spectra (left) or TEM images (right), scale bars = 200 nm. Representative images of two independent assemblies.

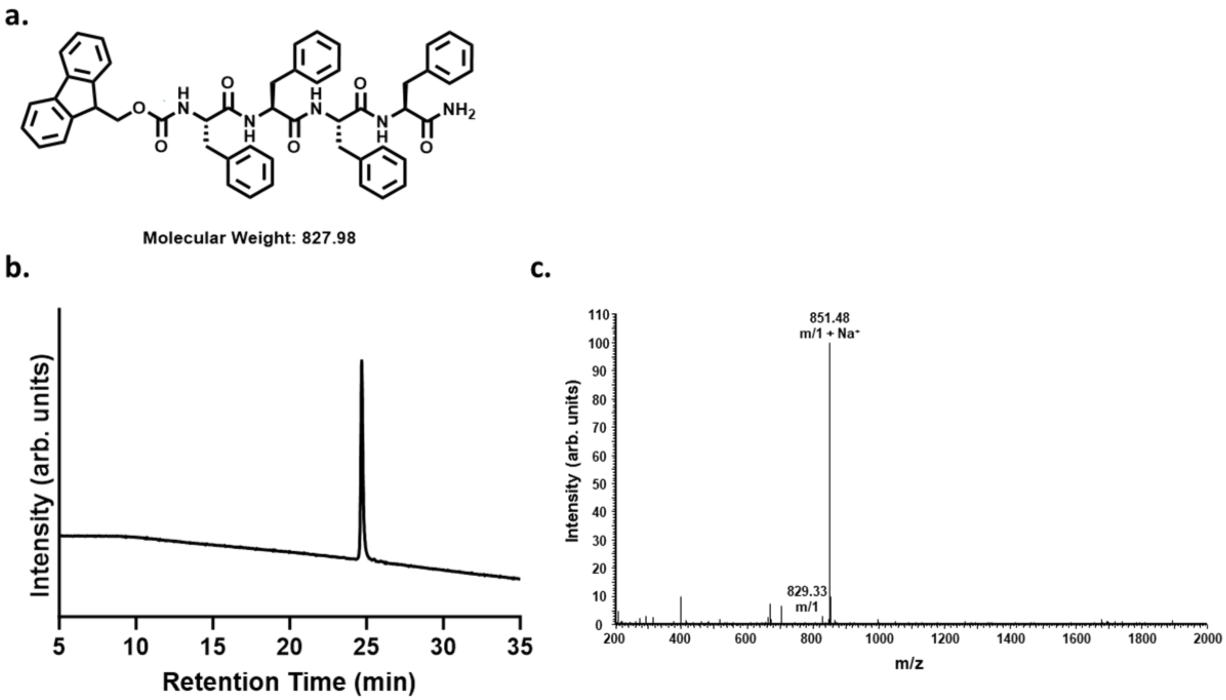

Supplementary Figure 26. Fmoc-FFFF-NH$_2$ Synthesis. Chemical structure (a) of the Fmoc-FFFF-NH$_2$ peptide. Analytical HPLC trace monitoring peptide absorbance at 214 nm (b) to confirm peptide purity and ESI mass spectrometry (c) confirming peptide identity.

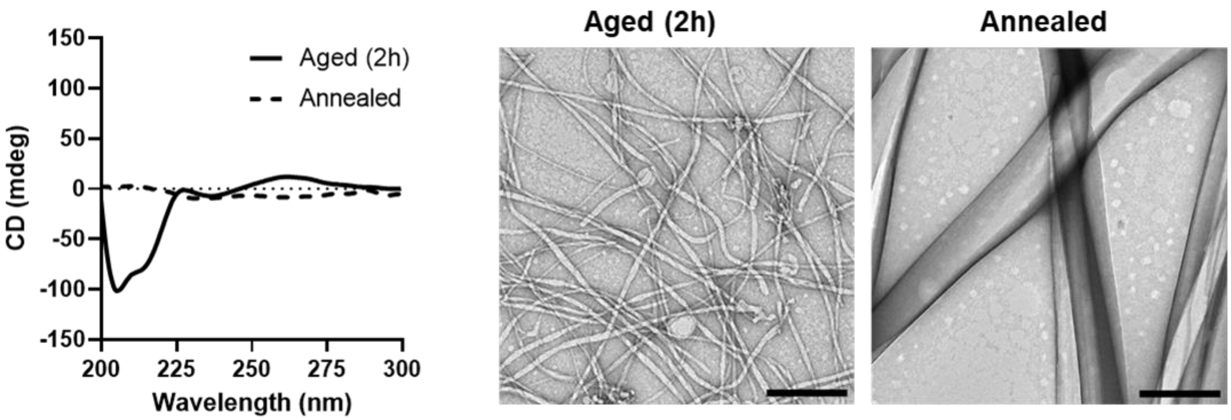

Supplementary Figure 27. Fmoc-FFFF-NH_2 CD and TEM. Fmoc-FF-PEG_2 was assembled in 10% HFIP for either 2h at room temperature or annealed before collecting CD spectra (left) or TEM images (right), scale bars = 200 nm. Representative images of two independent assemblies.

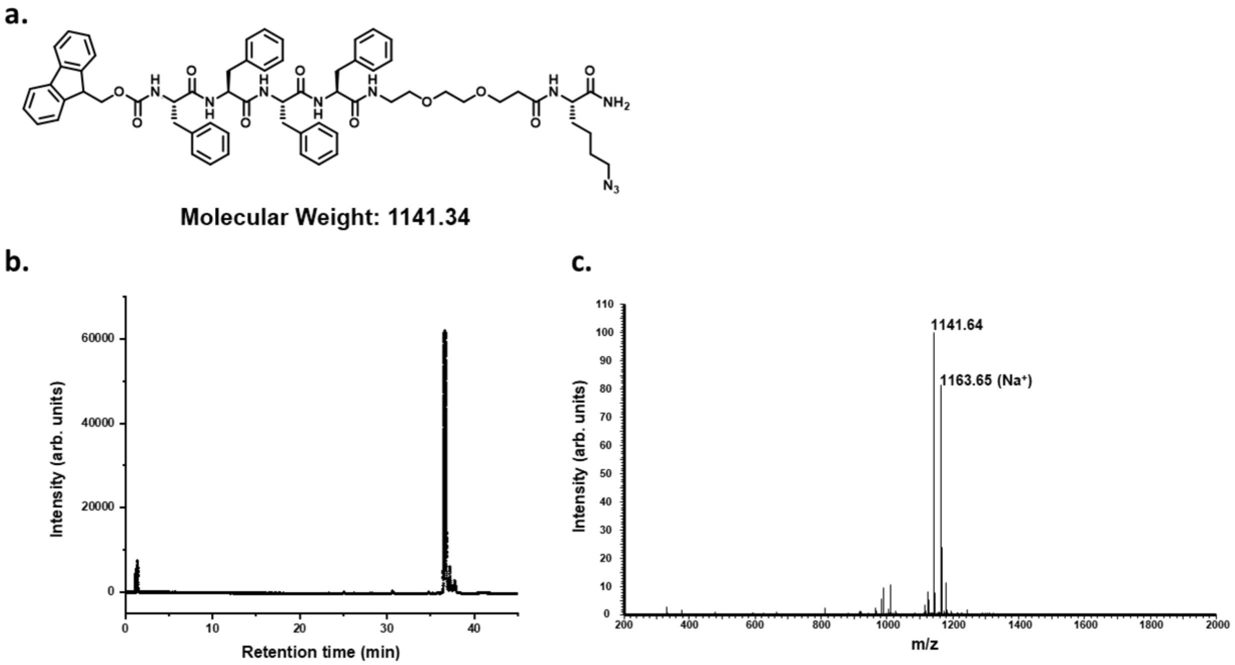

Supplementary Figure 28. Fmoc-FFFF-PEG_2-Kaz Synthesis. Chemical structure (a) of the Fmoc-FFFF-PEG_2-Kaz peptide. Analytical HPLC trace monitoring peptide absorbance at 214 nm (b) to confirm peptide purity and ESI mass spectrometry (c) confirming peptide identity.

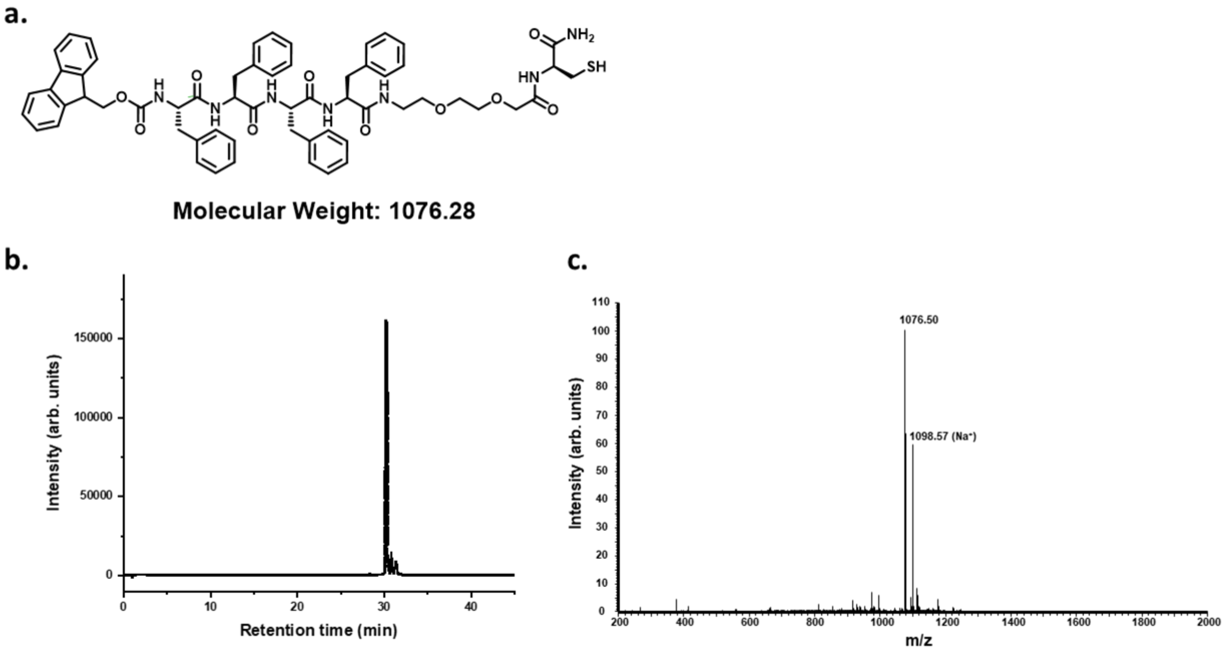

Supplementary Figure 29. Fmoc-FFFF-PEG$_2$-Cys Synthesis. Chemical structure (a) of the Fmoc-FFFF-PEG$_2$-Cys peptide. Analytical HPLC trace monitoring peptide absorbance at 214 nm (b) to confirm peptide purity and ESI mass spectrometry (c) confirming peptide identity.

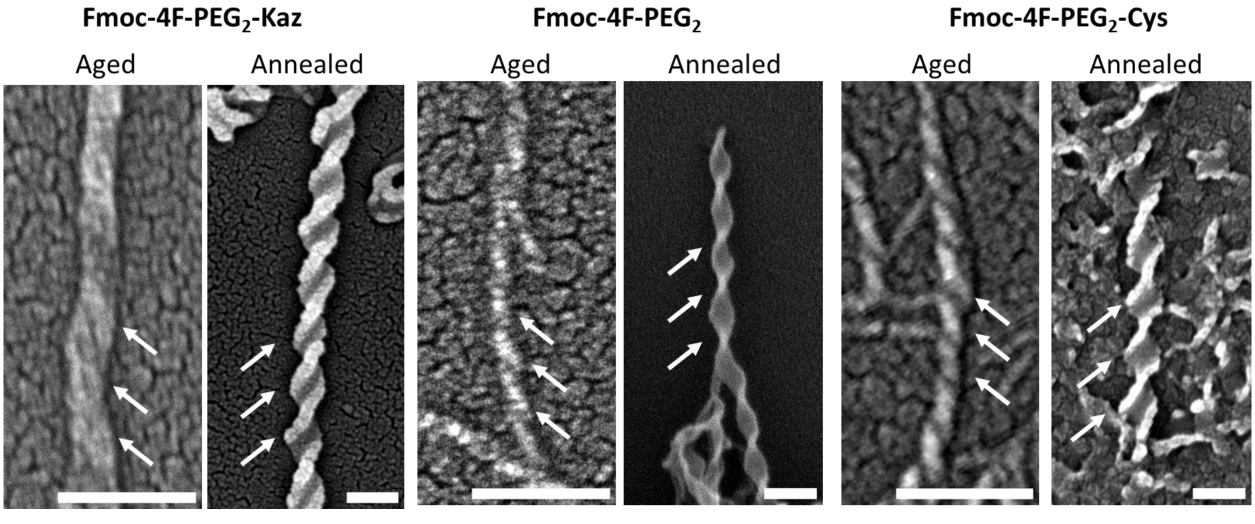

Supplementary Figure 30. SEM of Chiral Supramolecular Ribbons. Fmoc-FFFF-(R) samples were annealed at 250uM in 10% HFIP. 7.5 uL peptide solution was dried on a silicon wafer overnight at room temperature and coated with 4nm gold before imaging. White arrows denote the twist direction. Scale bars =100 nm. Representative images of two separate assemblies for each sample.

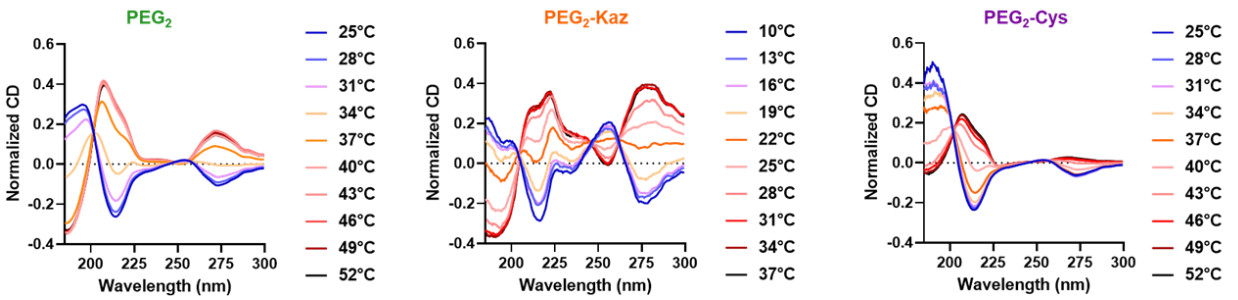

Supplementary Figure 31. Melting CD Spectra. CD spectra of Fmoc-4F-PEG2, Fmoc-4F-PEG2-Kaz, and Fmoc-4F-PEG2-Cys (left to right) during heating.

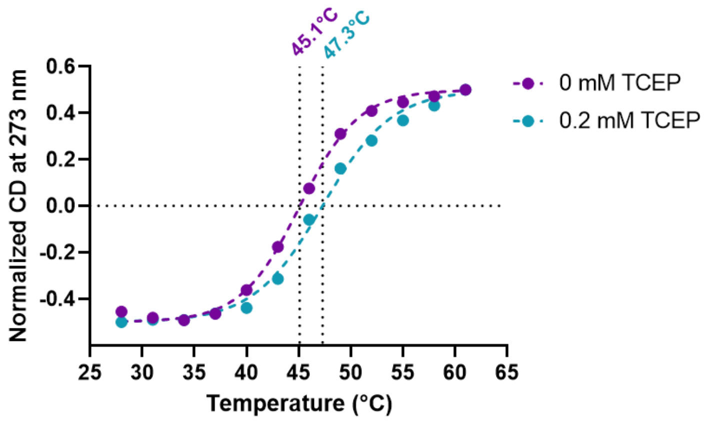

Supplementary Figure 32. Fmoc-4F-PEG2-Cys Melting with TCEP. CD melting curves of Fmoc-4F-PEG2-Cys assembled with and without 0.2 mM TCEP in 10% HFIP.

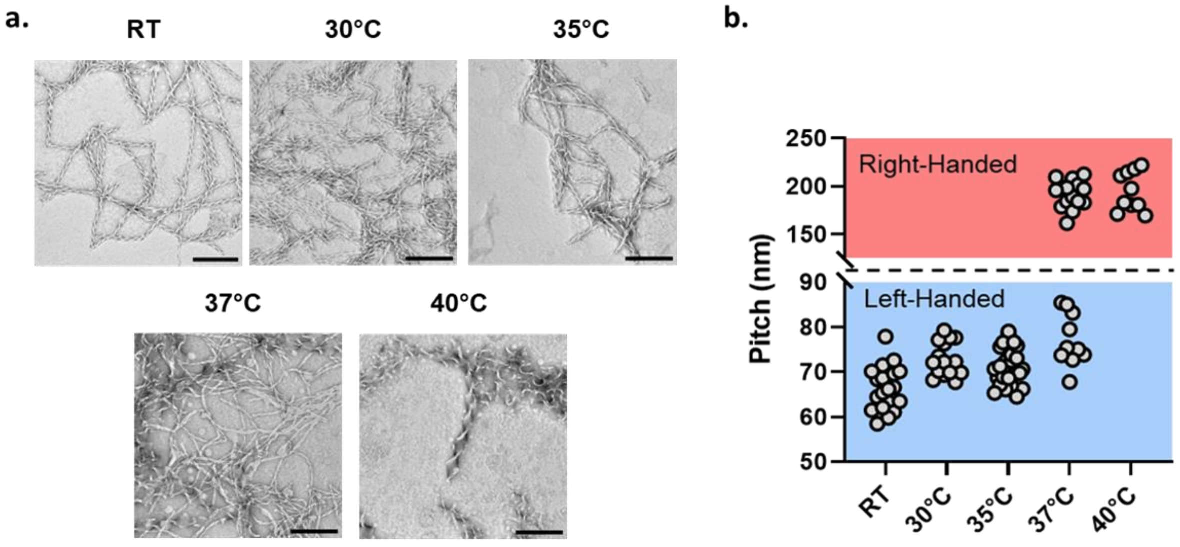

Supplementary Figure 33. Temperature Dependent Pitch of Fmoc-4F-PEG2. TEM images (a) at each temperature (scale bars = 200 nm). Pitch of twisted ribbons (b) was measured from TEM images of assemblies at each temperature (n= 20, 15, 25, 25, and 10 respectively). Representative images of a single sample for each temperature.

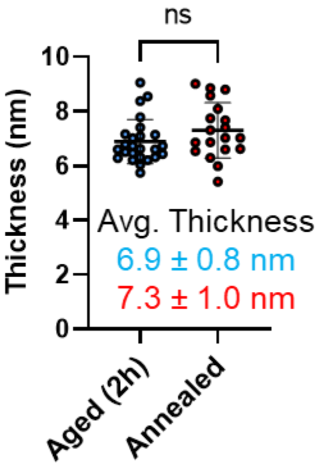

Supplementary Figure 34. Fmoc-4F-PEG$_2$ Ribbon Thickness. Ribbon thickness (on edge) was quantified from TEM images of aged and annealed Fmoc-FFFF-PEG$_2$ correlating to a peptide bilayer thickness (n=25, and 19 structures respectively). Mean and standard deviation of data points are shown with the error bars.

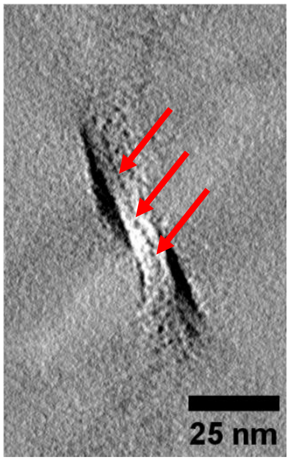

Supplementary Figure 35. Bilayer Assembly of Fmoc-4F-PEG$_2$. Slice of reconstructed tomogram of annealed Fmoc-4F-PEG$_2$ showing the bilayer of β-sheets within the ribbon. Representative of two independent reconstructions.

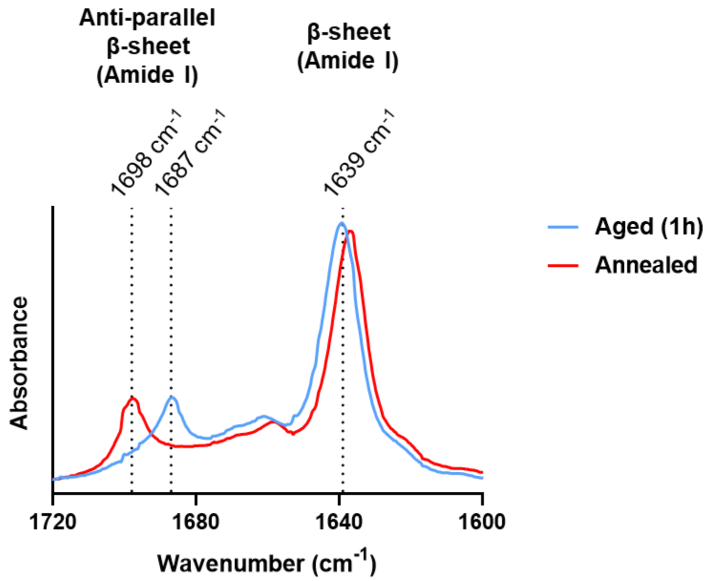

Supplementary Figure 36. FT-IR of Aged and Annealed Fmoc-4F-PEG$_2$. Solid-state ATR FT-IR spectra of dried Fmoc-4F-PEG$_2$ films, assembled in 10% HFIP then aged for 1 h (blue trace) or annealed (red trace). Spectra in the amide I region of peptide bonds (C=O stretching 1600-1700 cm^{-1}) show anti-parallel β-sheet signals (~1640 and ~1690 cm^{-1}). The annealed sample shows a larger higher frequency peak (1698) than the aged sample (1687), indicating possible changes between solvent-solute interactions that may be due to the expulsion of solvent molecules and tighter packing of hydrophobic moieties arising from heat treatment.

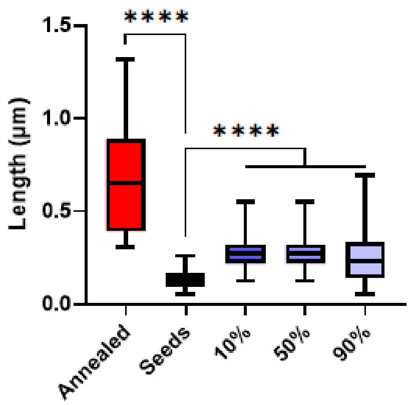

Supplementary Figure 37. Length of Nucleated Fmoc-4F-PEG$_2$. Length of right-handed assemblies found after annealing Fmoc-4F-PEG$_2$, after sonication to form seeds, and after 2 hours with varying ratios with unassembled peptide (n=15, 63, 52, 75, and 76 respectively). Center line is median, the box extends to the 25th and 75th percentiles, and whiskers extend to the minimum and maximum data points. Significance was determined with a one-way ANOVA and Tukey's multiple comparison test. Asterisks represent p values less than 0.0001. Annealed vs. Seeds, p=0.000000000000879, for seeds vs. the % seeds p values are 0.000000003416757, 0.000000003416757, 0.000002349238656 respectively.

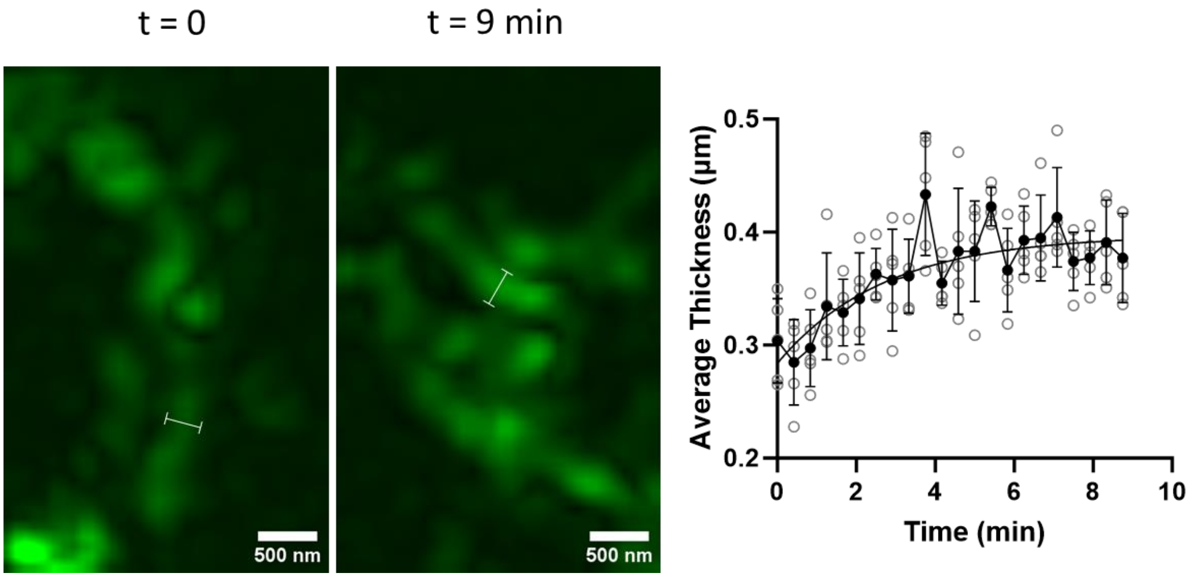

Supplementary Figure 38. Fmoc-4F-PEG$_2$ Structure Transition CLSM. Fmoc-4F-PEG$_2$ (aged) was diluted to 50μM (10% HFIP, 20% glycerol). The sample was stained with 10% ThT and placed on a heated confocal stage (60°C) the video was recorded as the sample warmed up and transitioned structures. Measurements of fiber thickness (n=5) at each time frame correspond to the formation of larger right-handed structures (n=5 structures measured at each time point). Mean and standard deviation are shown in the solid points; individual data points are shown with open circles. Green lookup table in ImageJ is used for displaying the images. Images and analysis were performed on a single video.

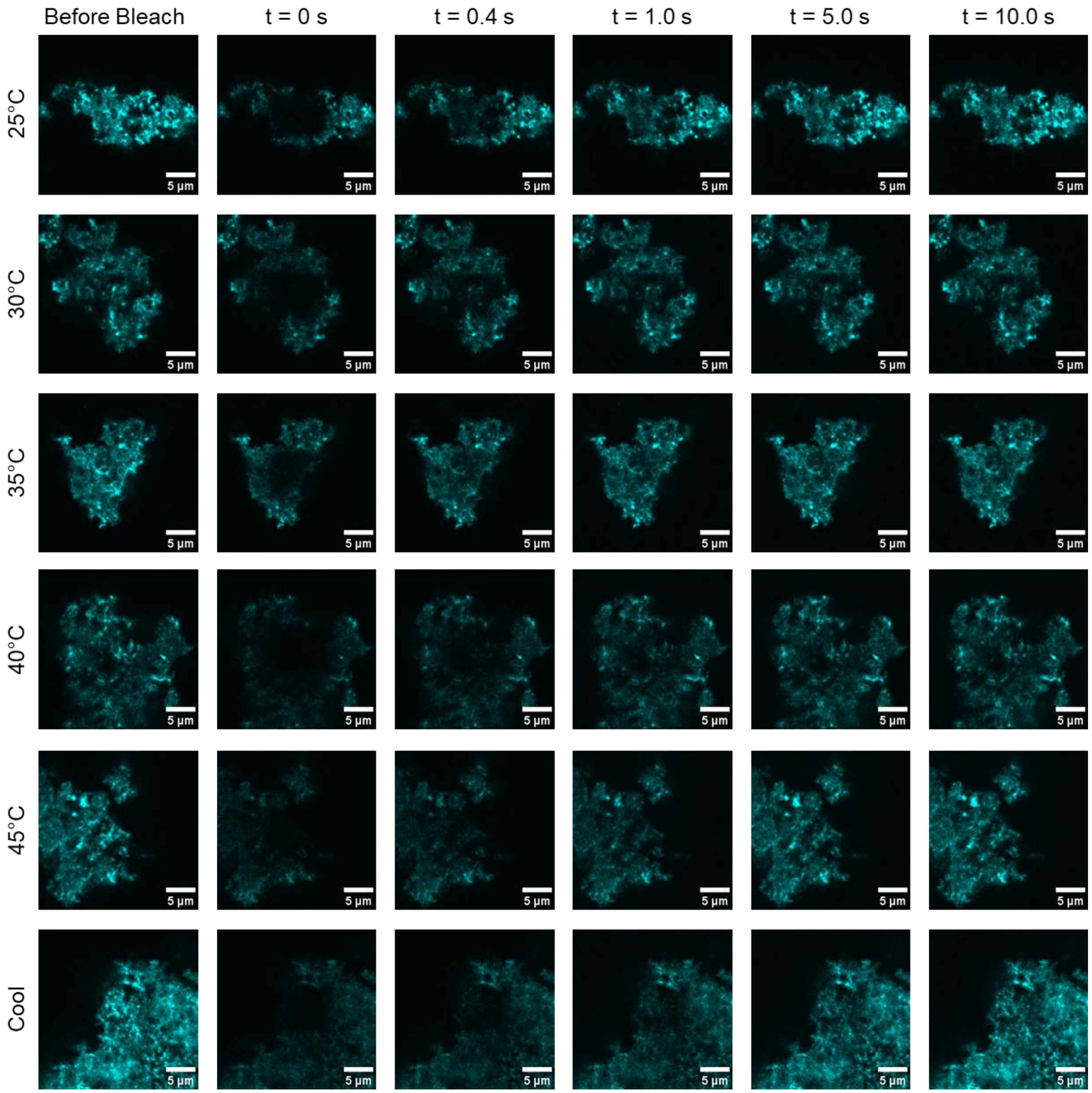

Supplementary Figure 39. Representative FRAP Images of Fmoc-FFFF-PEG$_2$. Fmoc-FFFF-PEG$_2$ was stained with ThT and heated on CLSM before acquiring FRAP videos at each temperature. Representative images from each temperature and selected time points are shown above (scale bar = 5µm). Cyan lookup table in ImageJ were used to display the images. Images are from a multiple positions for a single sample.

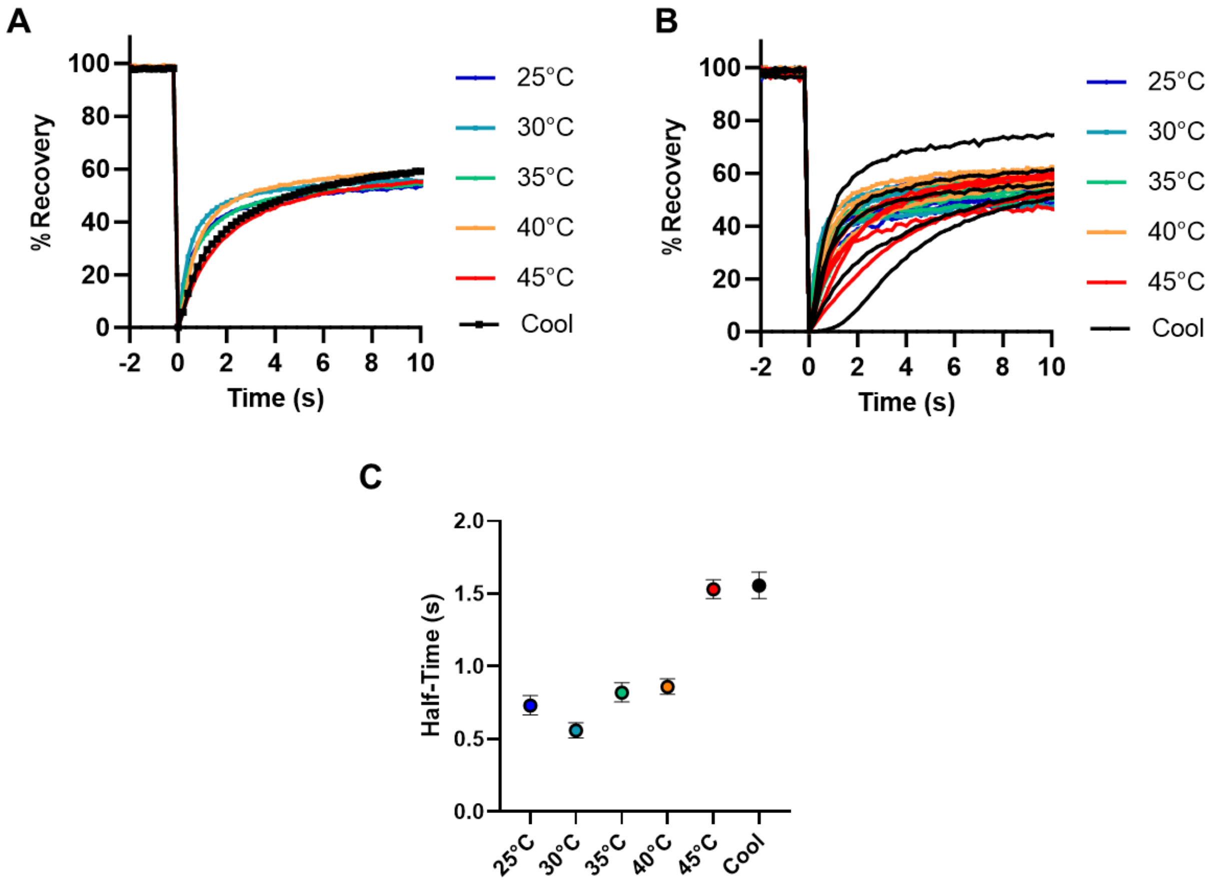

Supplementary Figure 40. FRAP Quantification of Fmoc-4F-PEG$_2$. Mean FRAP recovery curves (A) fitted to a one=phase association curve (n=5 videos). Individual curves are shown in (B). Half-life values (C) from FRAP recovery data shows slower recovery for the right-handed structures (data points shown are the half-life from fitting data in panel A with error bars showing the 95% confidence interval).

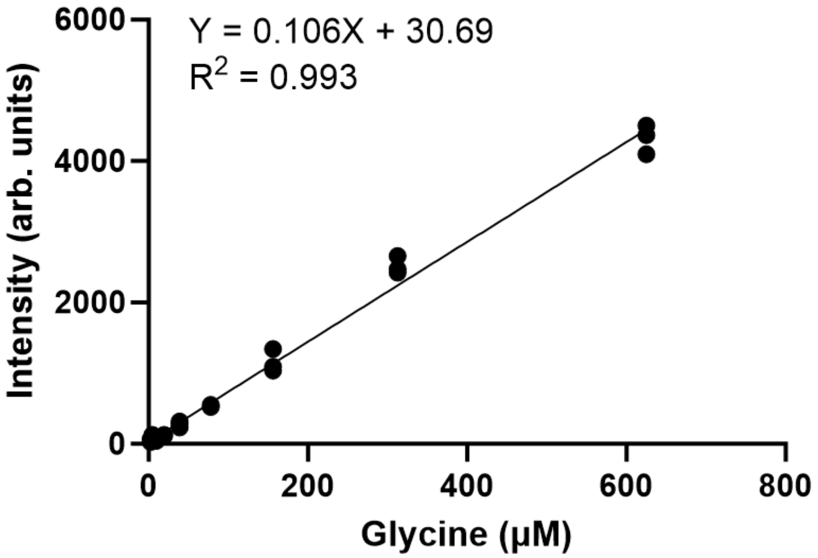

Supplementary Figure 41. Fluorescamine Standard Curve. Standard curve for fluorescamine assay calibration prepared with glycine standards (n=4 separate wells, all data points are shown). Curve fit: Y = 0.106X + 30.69.

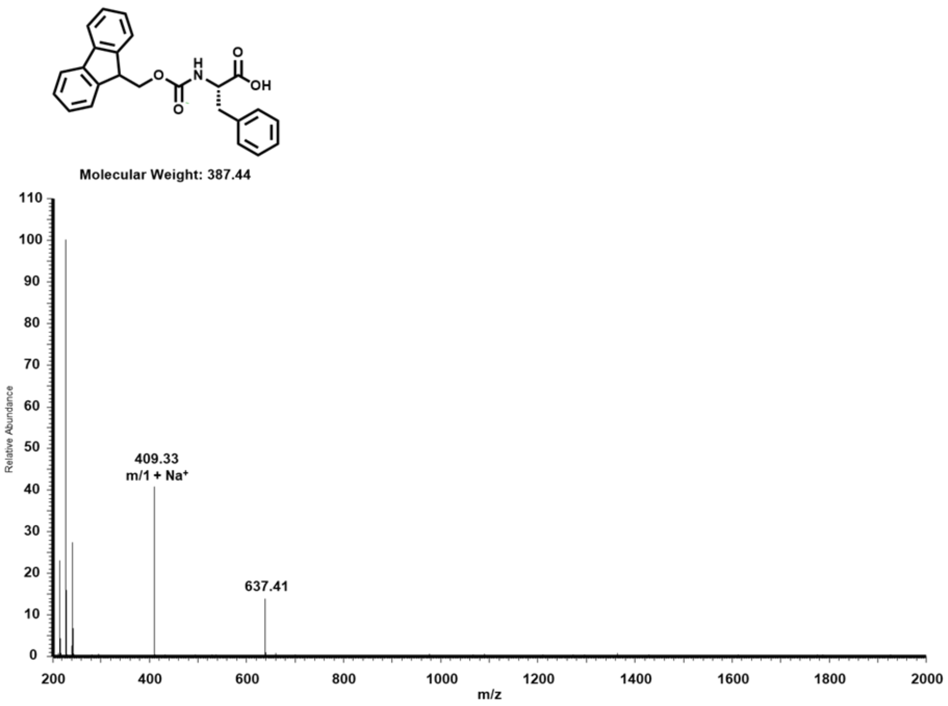

Supplementary Figure 42. Degradation MS 27 min. ESI+ mass spectrometry and chemical structure of identified product eluting at 27 minutes from the degradation analytical HPLC trace.

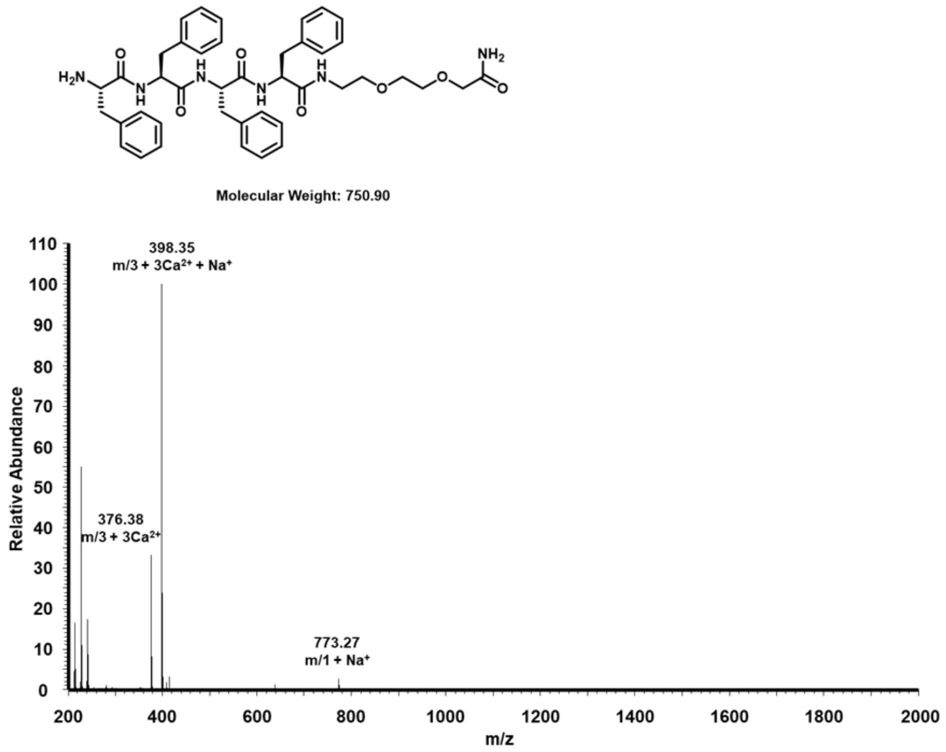

Supplementary Figure 43. Degradation MS 28 min. ESI+ mass spectrometry and chemical structure of identified product eluting at 28 minutes from the degradation analytical HPLC trace.

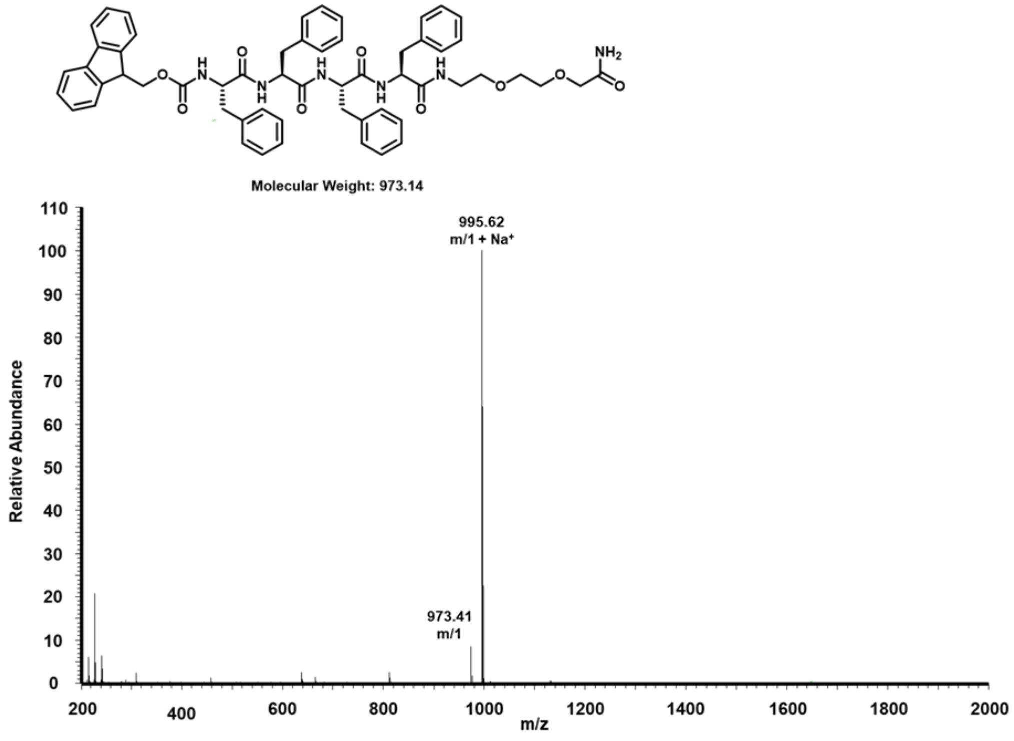

Supplementary Figure 44. Degradation MS 35 min. ESI+ mass spectrometry and chemical structure of identified product eluting at 35 minutes from the degradation analytical HPLC trace.

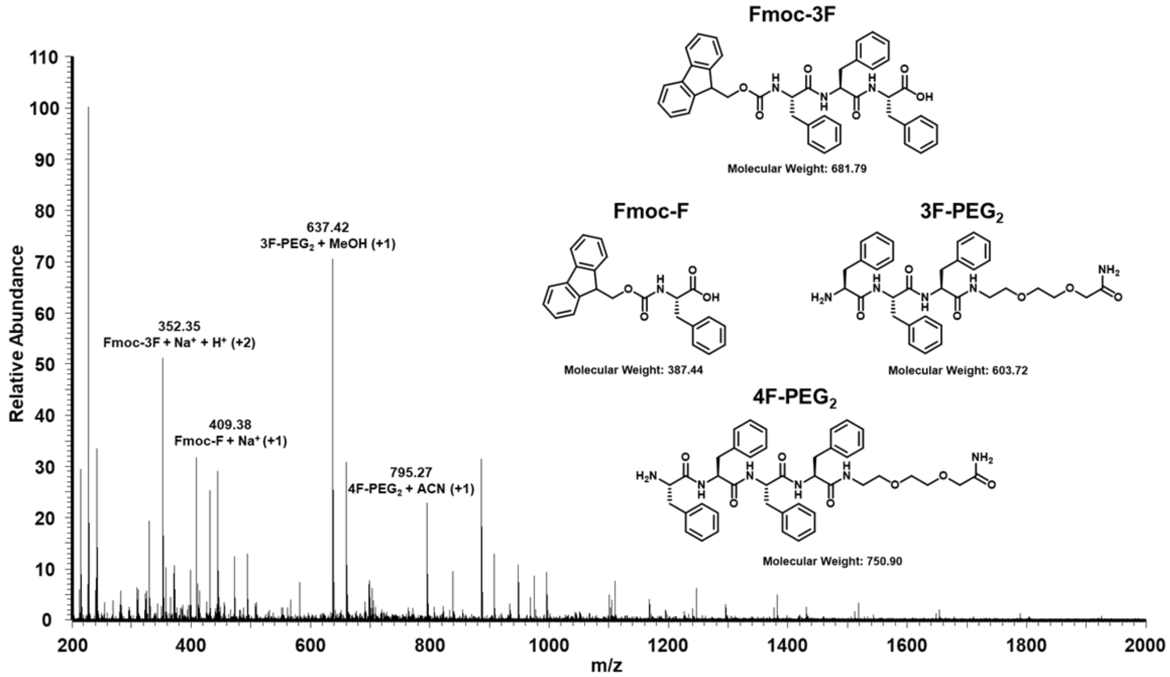

Supplementary Figure 45. Degradation MS 46 min. ESI+ mass spectrometry and chemical structure of identified products eluting at 46 minutes from the degradation analytical HPLC trace.

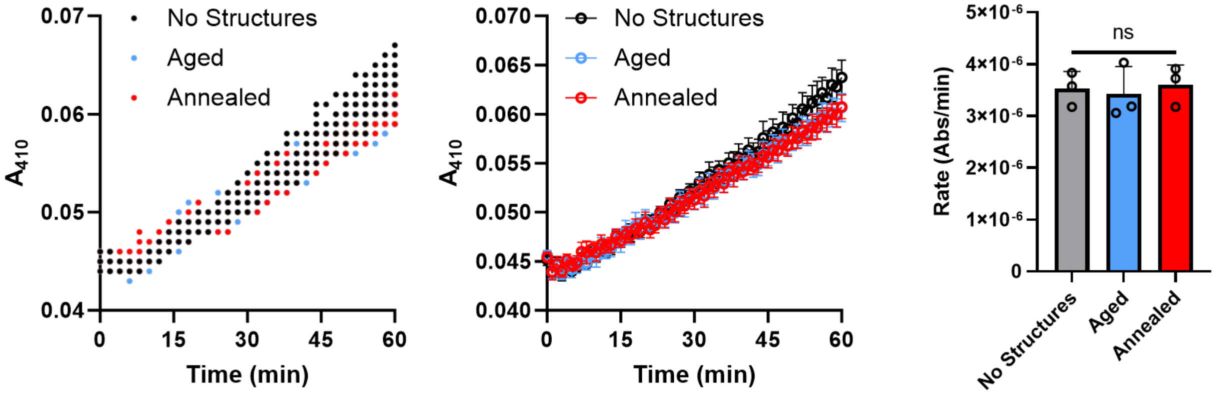

Supplementary Figure 46. Chymotrypsin Activity Assay. Chymotrypsin (200ug/mL) was used to digest left-handed (aged) and right-handed (annealed) Fmoc-FFFF-PEG$_2$ (10uM) for 24h at 30°C. After 24h, the remaining activity of enzyme was measured by the addition of 100 µM N-succinyl-L-phenylalanine-p-nitroanilide and absorbance was monitored at 410 nm (left shows individual data points, center shows mean and standard deviation since many data points are overlapping). This chymotrypsin activity assay shows that there is no significant reduction in enzyme activity after digesting either structure when analyzed with a one-way ANOVA ($p=0.8772$, $n=3$ separate assays for each case, mean and standard deviation show at each time point).

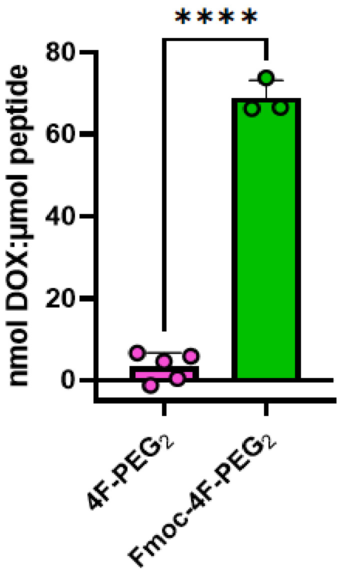

Supplementary Figure 47. DOX Loading for FFFF-PEG$_2$. DOX loading quantified from fluorescence comparing 4F-PEG$_2$ and Fmoc-4F-PEG$_2$ assembled at 250 µM in the presence of 50 µM DOX (n=5 and 3 samples of a single assembly of peptide centrifuged separately to collect DOX structures). Error bars shown represent the standard deviation of the data points from the mean (asterisks represent p=0.0000004 from an unpaired t-test).

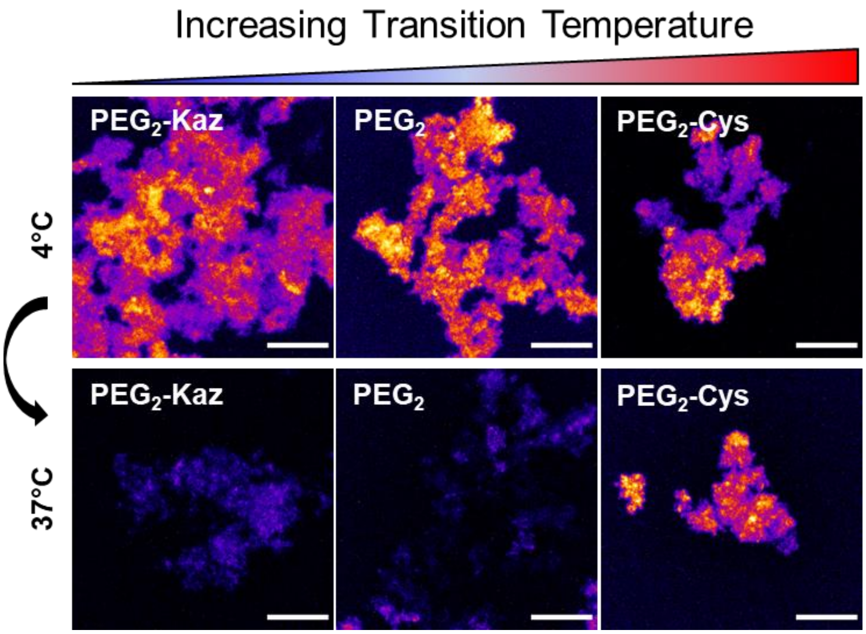

Supplementary Figure 48. CLSM of DOX Release. Peptides were assembled at 250 µM in the presence of 50 µM DOX in 10% HFIP. CLSM images were acquired from peptides assembled at 4°C before and after heating to 37°C for 1 hour (scale bars = 10 µm). Fire lookup table in ImageJ was used to display the images. Images a representative of 10 areas within a single sample for each peptide.

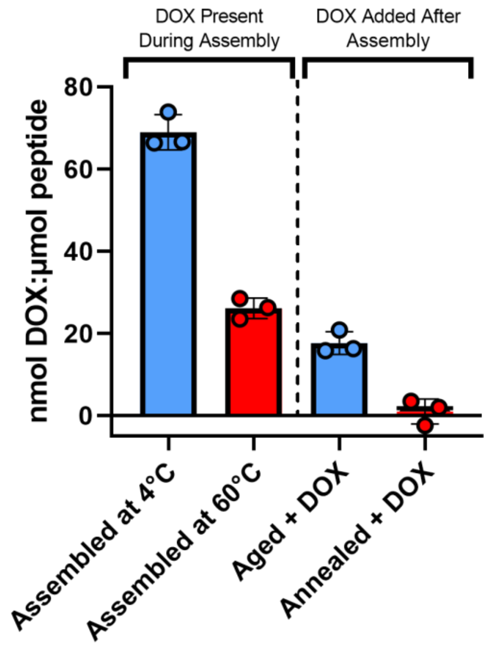

Supplementary Figure 49. DOX Binding to Left- and Right-Handed Structures. Fmoc-FFFF-PEG_2 was assembled at 250 µM in the presence of 50 µM DOX in 10% HFIP at either 4°C or 60°C with 50uM DOX to measure intercalation of left and right-handed states. Additionally, DOX (50uM) was added to pre-formed structures showing that adding DOX after assembly is less effective. In both cases DOX is seen to bind to the left-handed structures better. Error bars shown represent the standard deviation of the data points from the mean (n=3 samples of a single assembly of peptide centrifuged separately to collect DOX structures).

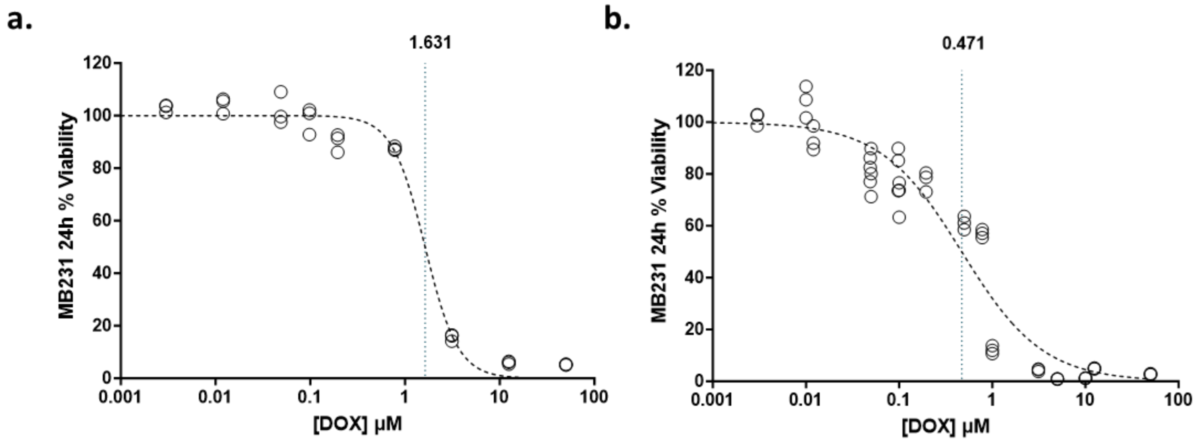

Supplementary Figure 50. DOX Calibration for HeLa Cells. Cell viability assays with free DOX at 24h (a) and 48h (b) with HeLa cells (n=3 wells for each treatment, all data points shown).

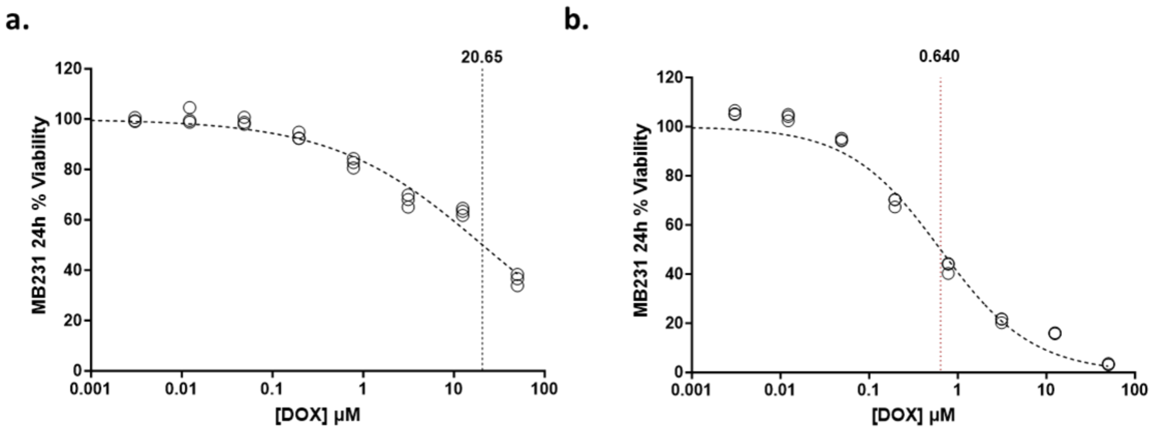

Supplementary Figure 51. DOX Calibration for MB231 Cells. Cell viability assays with free DOX at 24h (a) and 48h (b) with MB231 cells (n=3 wells for each treatment, all data points shown).

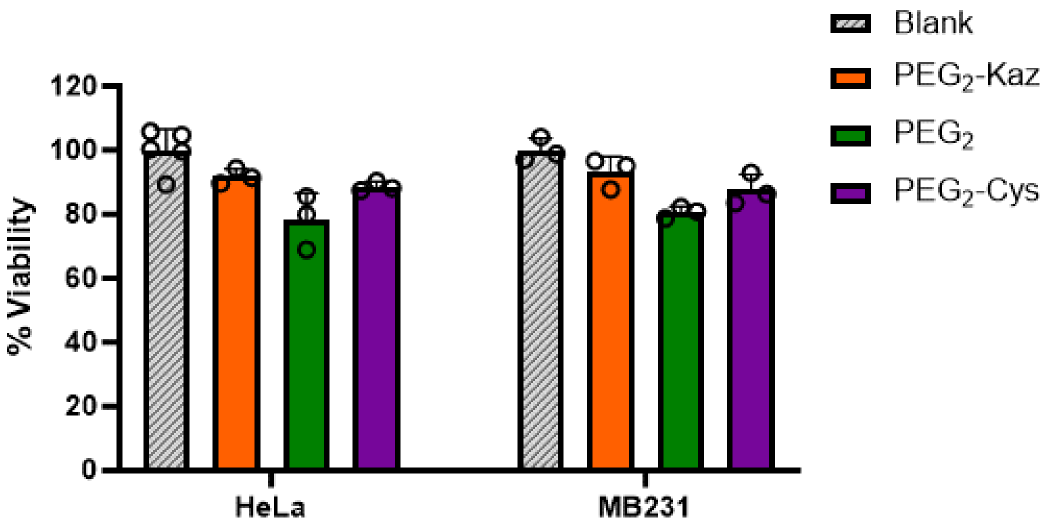

Supplementary Figure 52. Cell Viability at 24h. Cell viability results at 24h after the addition of DOX loaded peptides (n=3 wells for all samples except HeLa blank where n=5 wells). Error bars shown represent the standard deviation of the data points from the mean.

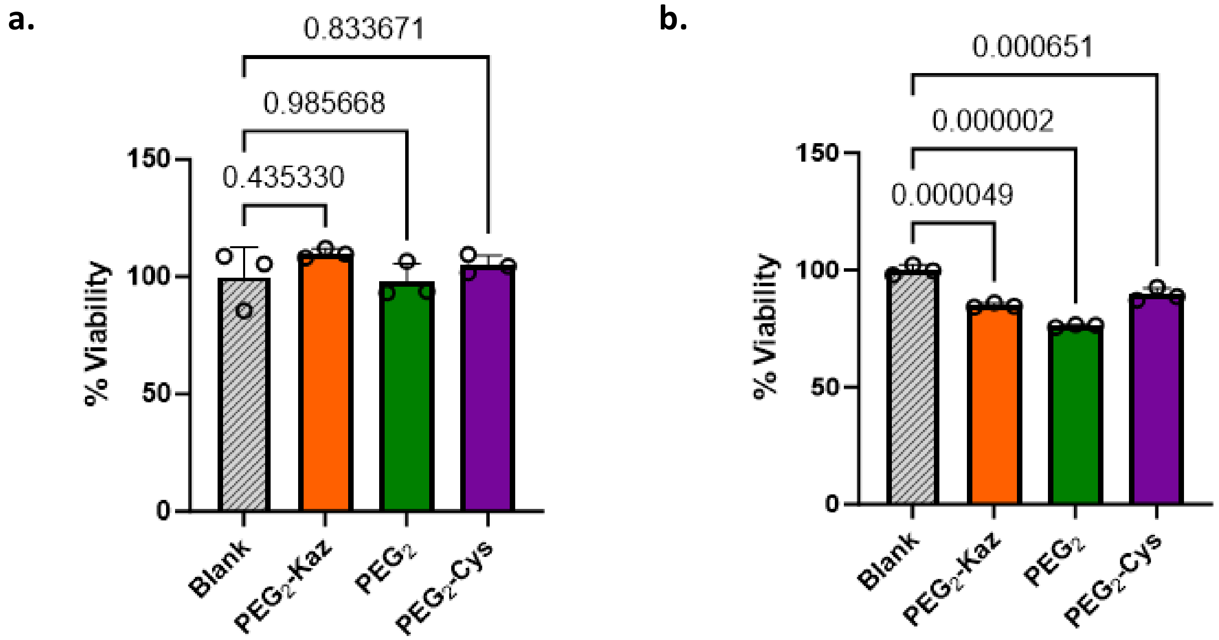

Supplementary Figure 53. Peptide Only Cell Viability Controls. Cell Viability results at 48 hours for peptides without DOX for Hela (a) and MB231 cells (b). Significance was determined with a one-way ANOVA and Tukey's multiple comparison test (n=3 wells, p values are shown on plot). Error bars shown represent the standard deviation of the data points from the mean.

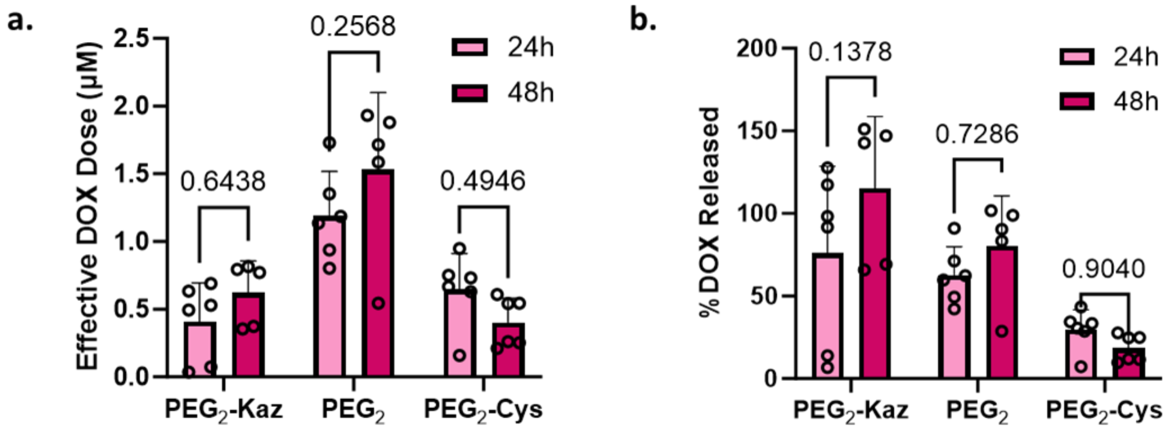

Supplementary Figure 54. Effective DOX Doses. Effective DOX doses released (a) and percentage of loaded DOX released (b) when incubated at 37°C. Values calculated from cell viability results. Significance was determined with a two-way ANOVA and Sidak's multiple comparison test. Error bars shown represent the standard deviation of the data points from the mean (n=6 wells of cell samples for all samples except 48h Kaz and PEG_2 samples where n=5 wells).

Supplementary References:

1. Mehta, A.K., Lu, K., Childers, W.S., Liang, Y., Dublin, S.N., Dong, J., Snyder, J.P., Pingali, S.V., Thiyagarajan, P., and Lynn, D.G. (2008). Facial Symmetry in Protein Self-Assembly. J. Am. Chem. Soc. *130*, 9829–9835. 10.1021/ja801511n.
