# Supplementary material for: Uncovering supramolecular chirality codes for the design of tunable biomaterials
Source: Nat Commun. 2024 Jan 26;15:788. doi: 10.1038/s41467-024-45019-2 (PMC10817930; doi:10.1038/s41467-024-45019-2)
Supplement: Supplementary file 3 — Description of Additional Supplementary Files [file 41467_2024_45019_MOESM3_ESM.pdf]

Description of Additional Supplementary Files

File Name: Supplementary Movie 1
Description: Electron Tomography Reconstruction Fmoc-FFFFPEG2. Movie of transmission electron tomography reconstruction of annealed FmocFFFF-PEG2 showing slices through the Z-axis after reconstruction in IMOD software. Scale bar = 50 nm

File Name: Supplementary Movie 2
Description: Confocal Video of Heating Fmoc-FFFF-PEG2 Network. Confocal movie of heating an aged Fmoc-FFFF-PEG2 network stained with ThT. Max intensity projection view and movie registration was performed in ImageJ. Scale bar = 5 µm.

File Name: Supplementary Movie 3
Description: Confocal Video of Heating Isolated Fmoc-FFFF-PEG2 Structures. Confocal movie of heating an aged Fmoc-FFFF-PEG2 sample (area with more isolated structures) stained with ThT. Max intensity projection view and movie registration was performed in ImageJ. Scale bar = 5 µm.
