# Supplementary material for: Uncovering supramolecular chirality codes for the design of tunable biomaterials
Source: Nat Commun. 2024 Jan 26;15:788. doi: 10.1038/s41467-024-45019-2 (PMC10817930; doi:10.1038/s41467-024-45019-2)
Supplement: Supplementary file 7 — Reporting Summary [file 41467_2024_45019_MOESM7_ESM.pdf]

nature portfolio

Corresponding author(s): David Lynn, Ronit Freeman

Last updated by author(s): Jan 1, 2024

Reporting Summary

Nature Portfolio wishes to improve the reproducibility of the work that we publish. This form provides structure for consistency and transparency in reporting. For further information on Nature Portfolio policies, see our Editorial Policies and the Editorial Policy Checklist.

Statistics

For all statistical analyses, confirm that the following items are present in the figure legend, table legend, main text, or Methods section.

n/a	Confirmed	
☐	☒	The exact sample size (n) for each experimental group/condition, given as a discrete number and unit of measurement
☐	☒	A statement on whether measurements were taken from distinct samples or whether the same sample was measured repeatedly
☐	☒	The statistical test(s) used AND whether they are one- or two-sided<br>*Only common tests should be described solely by name; describe more complex techniques in the Methods section.*
☐	☒	A description of all covariates tested
☐	☒	A description of any assumptions or corrections, such as tests of normality and adjustment for multiple comparisons
☐	☒	A full description of the statistical parameters including central tendency (e.g. means) or other basic estimates (e.g. regression coefficient) AND variation (e.g. standard deviation) or associated estimates of uncertainty (e.g. confidence intervals)
☐	☒	For null hypothesis testing, the test statistic (e.g. F, t, r) with confidence intervals, effect sizes, degrees of freedom and P value noted<br>*Give P values as exact values whenever suitable.*
☒	☐	For Bayesian analysis, information on the choice of priors and Markov chain Monte Carlo settings
☐	☒	For hierarchical and complex designs, identification of the appropriate level for tests and full reporting of outcomes
☒	☐	Estimates of effect sizes (e.g. Cohen's d, Pearson's r), indicating how they were calculated

Our web collection on statistics for biologists contains articles on many of the points above.

Software and code

Policy information about availability of computer code

Data collection	No code or software was used to collect data other than the software that each instrument is equipped with: Lab Solutions 5.92 (HPLC), LTQ Tune Plus 2.7 (Mass Spectrometry), SerialEM 4.1 and Digital Micrograph 3 (TEM), SkanIT 6.1 (Absorbance/Fluorescence), Chirascan 4 (CD), Zen Black 2.3 SP1 FP3 (confocal), IN Cell Analyzer 2200 version 7.2 (cell imaging).
Data analysis	FIJI ImageJ v1.54h and v1.53s, IMOD 4.11.24, and Graphpad Prism 9 and 10 were used for all data analysis.

For manuscripts utilizing custom algorithms or software that are central to the research but not yet described in published literature, software must be made available to editors and reviewers. We strongly encourage code deposition in a community repository (e.g. GitHub). See the Nature Portfolio guidelines for submitting code & software for further information.

Data

Policy information about availability of data

All manuscripts must include a data availability statement. This statement should provide the following information, where applicable:

- Accession codes, unique identifiers, or web links for publicly available datasets
- A description of any restrictions on data availability
- For clinical datasets or third party data, please ensure that the statement adheres to our policy

The spectral and imaging data generated in this study are available in the manuscript and supporting files. Data underlying plots is available in the source data file supplied with the manuscript. If other formats of the data generated during the current study are needed, they are available from the corresponding author upon request.

Research involving human participants, their data, or biological material

Policy information about studies with human participants or human data. See also policy information about sex, gender (identity/presentation), and sexual orientation and race, ethnicity and racism.

Reporting on sex and gender	No human participants or clinical data was used.
Reporting on race, ethnicity, or other socially relevant groupings	No human participants or clinical data was used.
Population characteristics	No human participants or clinical data was used.
Recruitment	No human participants or clinical data was used.
Ethics oversight	No human participants or clinical data was used.

Note that full information on the approval of the study protocol must also be provided in the manuscript.

Field-specific reporting

Please select the one below that is the best fit for your research. If you are not sure, read the appropriate sections before making your selection.

- [x] Life sciences
- [ ] Behavioural & social sciences
- [ ] Ecological, evolutionary & environmental sciences

For a reference copy of the document with all sections, see nature.com/documents/nr-reporting-summary-flat.pdf

Life sciences study design

All studies must disclose on these points even when the disclosure is negative.

Sample size	Predefined statistical sample size calculation was not used. Sample sizes were chosen to represent the observed variability within each group and to ensure reproducibility. These sample sizes yielded statistically significant ($p<0.05$) differences between groups. Specific sample sizes are provided for each experiment in the figure legends.
Data exclusions	All data was analyzed except in instances were samples were analyzed for curve-fitting or statistical significance, where a we utilized a predefined exclusion of outliers using the ROUT method with a Q=1% to enable more accurate analysis.
Replication	All attempts at replication were successful. For experiments repeated multiple times, the n is reported in the figure legends. When possible, multiple experimental techniques were used to validate a finding.
Randomization	For cell studies, all cultured cells of a given line were mixed prior to plating on assay plates to ensure all treatment groups have the same stock of cells so randomization of cell stocks was not implemented. Studies on pure synthetic peptides were taken from a purified stock for each peptide, so all studies for a given peptide were performed with identical materials so randomization of materials used was not needed. Within a sample, random locations were selected for imaging to provide an unbiased view.
Blinding	Blinding of samples was not performed as all conclusions reported are objective outcomes with quantitative results and there are no subjective outcomes measured that would be susceptible to researcher bias.

Reporting for specific materials, systems and methods

We require information from authors about some types of materials, experimental systems and methods used in many studies. Here, indicate whether each material, system or method listed is relevant to your study. If you are not sure if a list item applies to your research, read the appropriate section before selecting a response.

Materials & experimental systems

n/a	Involved in the study	
x		Antibodies
	x	Eukaryotic cell lines
x		Palaeontology and archaeology
x		Animals and other organisms
x		Clinical data
x		Dual use research of concern
x		Plants

Methods

n/a	Involved in the study	
x		ChIP-seq
x		Flow cytometry
x		MRI-based neuroimaging

Eukaryotic cell lines

Policy information about cell lines and Sex and Gender in Research

Cell line source(s)	MDA-MB-231 (ATCC HTB-26) is derived from a 51-year-old Caucasian female and was obtained from ATCC. HeLa cell line (ATCC CCL-2) is derived from a 31-year-old Black female and was obtained from the UNC Tissue Culture Facility.
Authentication	No authentication was performed after purchasing. Prior to purchase, cell lines were authenticated commercially by ATCC using STR profiling.
Mycoplasma contamination	Cell lines were not tested for mycoplasma contamination after purchase. Prior to purchase, cell lines were verified as mycoplasma negative by ATCC.
Commonly misidentified lines (See ICLAC register)	No commonly misidentified cell lines according to the ICLAC registry were used in this study.
